# Cellular Responses to Mechanical Cues Across Scales: From Fundamental Insights to Translational Potential

**DOI:** 10.1002/adhm.71400

**Published:** 2026-07-06

**Authors:** Mathias Polz, Manuel P. Kainz, Niklas Haider, Theresa Rienmüller, Kerstin Lenk, Rupert Kargl, Michele Terzano, Gerhard Sommer, Gerhard A. Holzapfel, Julia Fuchs

**Affiliations:** ^1^ Institute of Biomechanics Graz University of Technology Graz Austria; ^2^ Department of Neurosurgery Medical University of Graz Graz Austria; ^3^ BioTechMed‐Graz Graz Austria; ^4^ Institute of Neural Engineering Graz University of Technology Graz Austria; ^5^ Institute for Chemistry and Technology of Biobased Systems (IBioSys) Graz University of Technology Graz Austria; ^6^ European Polysaccharide Network of Excellence (EPNOE) Leuven Belgium; ^7^ Department of Structural Engineering Norwegian University of Science and Technology (NTNU) Trondheim Norway; ^8^ Division of Cell Biology Histology and Embryology Gottfried Schatz Research Center Medical University of Graz Graz Austria

**Keywords:** cell–material interactions, cellular microenvironment, mechanobiology, mechanotransduction, multiscale in vitro models, soft biomaterials, soft tissue biomechanics, translational tissue models

## Abstract

Soft biomaterials strongly influence cellular behavior by transmitting mechanical cues via well‐characterized mechanotransduction pathways. However, translating insights from simplified in vitro systems to complex in vivo responses remains a challenge. This review provides a framework for assessing the translational relevance of mechanobiological studies across different scales—from single cells and organoids to tissues and organs. First, we categorize soft biomaterials based on their architecture and mechanical features to establish consistent terminology. We then investigate how cells interpret the transmitted mechanical signals in advanced in vitro systems that simulate physiologically relevant mechanical environments. To discuss the translational potential, we present a scoring system and a comparative analysis demonstrating that physiological specificity, rather than experimental complexity, determines predictive value. Simple systems can yield highly translatable outcomes when mechanical cues mimic native conditions, while advanced models require careful validation to ensure their relevance. By integrating biomechanics, electrophysiology, and systems biology, we outline principles and validation strategies that enhance the predictive utility of mechanobiological findings and ultimately support the design of more effective biomaterials and tissue‐engineered systems.

## Graphical abstract

This review examines how cellular behavior is regulated by mechanical cues transmitted through soft biomaterials, from single‐cell mechanosensing to tissue‐level adaptation. It highlights why physiological relevance, rather than model complexity alone, is critical for translational mechanobiology and introduces a scoring framework linking material properties, mechanotransduction, and multiscale validation to improve prediction of in vivo outcomes.

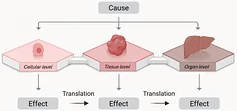

## 1 Introduction

Current paradigms in soft implants and three‐dimensional (3D) in vitro models focus on replicating the properties of biological tissues or environments by utilizing synthetic materials that guide cellular behavior or integrate seamlessly within biological systems [1, 2]. While biochemical interactions—such as protein adsorption and enzymatic activity—play essential in modulating force transmission, the mechanical properties and structural parameters, including specific microarchitecture and material properties of a biomaterial, directly dictate the cell behavior. Since virtually all cells in the human body respond to mechanical cues, the strategic modulation of these environments can be used to influence cell function, adhesion, proliferation, differentiation, and morphogenesis, as well as the ultimate biomaterial integration [3, 4, 5, 6]. Importantly, these mechanically regulated processes occur at the cellular level, e.g., molecular force sensing at adhesion complexes, but eventually impact tissue remodeling and organ‐level function. Consequently, mechanobiology is inherently a *multiscale* discipline, where local cellular responses can propagate into emergent multicellular and physiological outcomes. More specifically, structural features and mechanical properties of biomaterials act as upstream regulators of mechanotransduction by influencing alignment, migration, and spreading of cells [7]. Therefore, advanced biomaterial design can be used to mimic healthy physiological processes or modulate pathological tissue microenvironments. This multiscale coupling is particularly relevant for translational biomaterials, since material properties engineered at the nano‐ or micro‐level can ultimately influence macroscopic tissue integration, regeneration, fibrosis, or implant failure in vivo.

**FIGURE 1:**
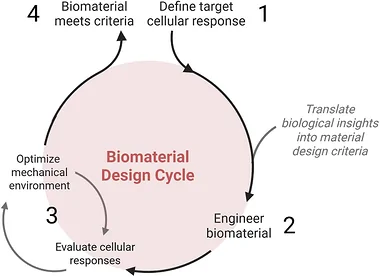
Biomaterial Design Cycle. The workflow begins with the definition of a desired cellular response (1), informed by biological questions and relevant in vivo or physiological conditions. These biological insights are translated into qualitative and quantitative material design criteria, which guide the engineering of the biomaterial (2). The engineered biomaterial is then evaluated through in vitro or ex vivo assays to characterize cellular responses such as adhesion, signaling, differentiation, or functional output (3). Based on these results, the mechanical environment is systematically optimized and refined. The cycle is then completed when the biomaterial meets predefined biological and performance criteria (4), at which point the design can be validated or further iterated to improve fidelity, tunability, and physiological relevance.

In biomaterial engineering, a fundamental distinction exists between *biomimetic biomaterials*, designed to replicate native biological environments, and *tunable biomaterials*, which actively guide target cellular behavior through programmed mechanical stimuli [8, 9, 10]. In the first category, tissue‐mimicking materials or materials mimicking the extracellular matrix (ECM) of soft tissues incorporate features defined by the physiological conditions cells experience in vivo. In contrast, tunable biomaterials are engineered to achieve specific cellular responses through targeted cues. These biomaterials typically undergo iterative optimization, as outlined in the *Biomaterial Design Cycle* (Figure 1). Crucial to this design process is the validation of the response, which ensures that the biomaterial modulates cell behavior as desired. This forms the basis for translatable experimental setups and context‐dependent insights. Ultimately, this design process increases the reliability of in vitro models, advances material engineering, and supports clinical translation [11, 12, 13, 14].

Inspired by native biological environments, in vitro models utilizing synthetic biomaterials as substrates or scaffolds provide valuable mechanistic insights into cell‐material interactions and cellular mechanotransduction pathways, while reducing reliance on animal models [13, 15, 16, 17]. These systems serve as controllable platforms for dissecting causal mechanotransduction pathways at the cellular level. However, successful translation requires understanding how these responses scale toward collective tissue behavior and organ physiology. Cells in vivo exist within dynamic multicellular environments in which mechanical forces are transmitted across interconnected molecular, cellular, tissue, and organ scales. Mechanical signals are spatially heterogeneous, temporally evolving, and continuously reshaped by reciprocal interactions between cells, ECM, immune populations, and physiological loading conditions. In contrast, in vitro systems are engineered to provide a high degree of experimental controllability, prioritizing the isolation of specific variables and mechanistic pathways. Therefore, the primary translational challenge lies not in unpredictability, but in the difficulty of capturing the coupled, multi‐factorial nature of physiological systems within controlled experimental frameworks. While advanced 3D culture systems, such as organoids and engineered tissues, increasingly incorporate dynamic features, many in vitro models remain simplified static mechanical environments. Native tissues are exposed to complex combinations of mechanical stimuli, including oscillatory forces, cyclic strain, and dynamic interstitial fluid flow [18, 19], which are often only partially recapitulated in vitro. Consequently, the mechanotransduction pathways regulating cell differentiation, migration, and tissue remodeling may be differentially activated, leading to discrepancies in cellular behavior and responses between in vitro and in vivo settings [20, 21]. This trade‐off between controllability and physiological complexity represents both a fundamental limitation and a key design challenge in translational mechanobiology. As a result, many mechanobiological findings remain valid at the single‐cell or local tissue scale but fail to fully predict long‐term in vivo responses, where immune modulation, vascularization, remodeling, and systemic feedback mechanisms become dominant.

Furthermore, considering inherent material limitations is essential for successful biomaterial engineering. For example, a significant mechanical mismatch between abiotic materials and the host's biotic environment in vivo can trigger unwanted cellular responses and immune reactions, culminating in implant rejection, impaired cellular function, and a cascade of pathological effects, including chronic inflammation, fibrosis, and the formation of apoptotic tissue [22]. These immune responses and inflammatory signaling pathways, which are crucial in determining implant success, are often absent or altered in in vitro models, further complicating the prediction of in vivo integration [19, 23]. Another critical factor affecting translatability is that artificial scaffolds typically lack the complex, hierarchical architecture and dynamic, adaptive behavior of the native ECM. This structural simplicity directly impacts cellular mechano‐sensing and long‐term adaptability, hindering cell adhesion and tissue integration [21, 24].

Bridging the gap between results obtained in simplified in vitro models and the complex tissue reactions observed in vivo requires improved experimental designs and highly physiologically relevant models [25, 26]. The integration of dynamic mechanical stimuli, such as those provided by flow bioreactors, alongside immune‐competent co‐culture systems, enables a better prediction of in vivo outcomes and the reproduction of physiological mechanotransduction pathways [27, 28, 29]. Many of these methodologies require specialized expertise and advanced infrastructure, often facing challenges in experimental reproducibility. Therefore, simpler cell culture models remain prevalent despite their limited translatability, with multiple experimental setups combined to offset individual shortcomings. Ultimately, the successful translation of in vitro findings to in vivo outcomes requires not only the systematic use of complementary approaches but also a rigorous understanding of causal relationships. This includes distinguishing the primary effects of specific material parameters from indirect or secondary responses, such as those mediated by downstream signaling pathways or immune activation, to ensure physiological relevance in line with the respective research question.

**FIGURE 2:**
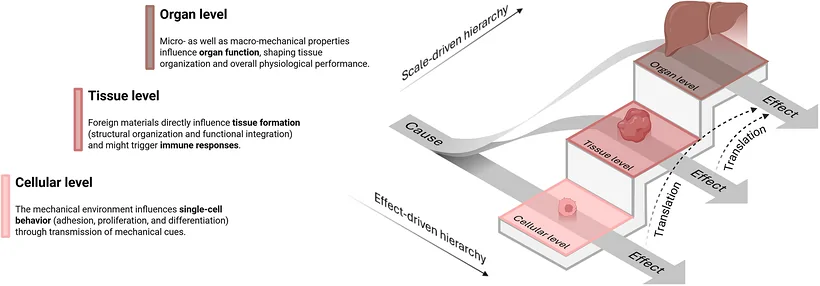
Framework to evaluate translational potential of in vitro models. A multidimensional cascade illustrates the progression from the initial cause (mechanical input) to the effect (physiological response) across a scale‐driven hierarchy: cellular level (influencing single cell behavior), tissue level (influencing tissue formation), and organ level (influencing organ function).

In this review, we first categorize biomaterials and discuss their fundamental properties, followed by an overview of state‐of‐the‐art cell culture systems designed to replicate in vivo mechanical dynamics. To evaluate the translational potential of experimental setups and outcomes, we employ two distinct analytical frameworks (Figure 2): an *effect‐driven* pathway, which traces mechanotransduction from initial mechanical input to downstream cellular and physiological responses, and a *scale‐driven* hierarchy, which examines how mechanical cues are integrated across single cells, tissues, and organs. These hierarchies illustrate how mechanical environments dictate biological outcomes at varying levels of complexity. Notably, they highlight that while specific mechanical cues can trigger similar cellular responses at the single‐cell level, in tissue or organ contexts, emergent, scaledependent behaviors and complex cell‐cell interactions can lead to entirely different feedback‐driven effects.

This overview also underscores that the interplay of material properties and structural architecture—from the micro‐ to macro‐level—exerts a differential influence on cellular behavior. Accordingly, translational mechanobiology requires experimental systems that not only reproduce isolated mechanical parameters but also preserve the cross‐scale coupling between cellular mechanosensing, multicellular coordination, tissue remodeling, and physiological function. By integrating insights from biomechanics, electrophysiology, and biochemistry, we propose a unified framework for understanding mechanotransduction and its context‐dependent translational potential. Furthermore, we investigate correlations between different studies to identify and highlight common, often‐overlooked limitations hindering the field's progress. Finally, this analysis is intentionally restricted to soft biomaterials; by narrowing our scope to their specific architectures, mechanical responses, and specialized experimental methods, we ensure a more efficient and cohesive presentation.

## 2 Soft Biomaterials and Their Mechanical Environment

The design of soft biomaterials for tissue engineering and mechanobiological applications requires a comprehensive understanding of how the *mechanical environment* influences cellular responses across scales. In this context and throughout this work, *soft* refers to reduced *stiffness* (see Box 1) in the range of a few pascals to several tens of kilopascals, corresponding to the mechanical window of numerous biological tissues and biomaterials [2, 30].

This section aims to provide an introductory, structured guide to assess soft biomaterials as the mechanical environment of cells, laying the foundations for the subsequent analyses. In mechanobiology, the term *mechanical environment* encompasses not only the magnitude and type of mechanical stimuli acting on cells—such as stiffness, viscoelasticity, shear stress, compression, cyclic stretch, osmotic pressure, or confinement—but also the spatial context in which these forces are experienced. Cellular mechanosensing is inherently dependent on tissue architecture, including whether cells are cultured as isolated single cells, confluent monolayers, co‐cultures, spheroids, organoids, or within 3D ECMs. Consequently, architectural organization and mechanical parameters cannot be considered independently in biological model systems. The same mechanical stimulus can induce fundamentally different cellular responses depending on dimensionality, cell‐cell contact, matrix composition, or multicellular coordination.

For this reason, this review first introduces terminology describing experimental architecture that later defines model complexity before discussing specific mechanical loading paradigms and biomaterial systems in greater detail. Establishing this framework is essential because the interpretation, physiological relevance, and translational potential of mechanobiological findings depend on the interplay between initial material compatibility, biomaterial mechanics, and the structural organization of the in vitro model. This perspective allows for detailed analysis of cellular responses and physiological relevance depending on the whole environment and not single parameters.

Biocompatibility is context‐dependent and describes the ability of a material to elicit an appropriate host response in a specific application [31, 32]. In mechanobiology, it comprises interconnected cellular, immunological, chemical, and mechanical dimensions that together determine the physiological relevance and translational potential of experimental findings. In vitro compatibility is commonly assessed through viability and metabolic assays, whereas in vivo compatibility also includes immune responses and degradation‐related effects that can directly influence mechanotransduction and long‐term tissue integration.

While cytocompatibility, immunological safety, and chemical stability are fundamental prerequisites for translational research, the concept of *mechanocompatibility* is the core dimension of this work. Mechanocompatibility defines the degree to which a material's mechanical environment effectively guides and supports desired cellular behaviors. This encompasses both intrinsic properties and the extrinsic forces, such as shear or tensile loading, that cells experience within the matrix [33]. For instance, tunable hydrogel matrices that mimic tissue‐level stiffness and viscoelasticity can enhance attachment and direct stem cell differentiation by providing high‐fidelity mechanical cues [34, 35]. Moreover, mechanocompatibility is shaped by bidirectional biomaterial‐cell interactions, in which cells adapt to local mechanical cues while simultaneously remodeling or degrading the biomaterial environment over time. Consequently, assessing mechanocompatibility and mechanoadaptation alongside biochemical traits is essential for ensuring the physiological relevance of in vitro models.

Notably, mechanocompatibility should not be viewed as an intrinsic characteristic of the biomaterial itself but rather as an emergent property that depends strongly on cellular context and organization. In addition to spatial organization, the relevant time scales of loading and matrix mechanics (e.g., viscoelastic dissipation, stress relaxation, and dynamic stiffening/softening) can critically alter mechanosensing and downstream signaling. Therefore, we propose a score that explicitly includes these contextual factors and enables comparability across different cell types and organizational levels (see section 7.2).

To evaluate mechanocompatibility, we first need to identify the aspects of a biomaterial's mechanical environment, including specific architectural features alongside mechanical parameters. Therefore, we begin with a phenomenological categorization of biomaterials based on architecture and geometrical features. In this context, *phenomenological* means that observable parameters, specifically geometrical features, are prioritized over intrinsic parameters such as chemical composition. The assessment of relevant mechanical parameters is not trivial, and commonly used concepts such as elastic modulus, shear modulus, and loss modulus are treated as intrinsic material properties, although they are actually phenomenological parameters. To illustrate how cells perceive their mechanical environment, we provide an overview of the fundamental modeling approaches to describe mechanical behavior and reference selected works that provide relevant applications to soft biomaterials.

**BOX 1 ‐ Stiffness: Definition and Limitations as a Material Parameter**

In materials science, stiffness refers to the resistance of a material to deformation when a load is applied. The term is used in two distinct but related ways. First, it may denote an *intrinsic material property*, determined under controlled, homogeneous deformation modes and within a limited strain range. Here, it is essentially synonymous with an elastic modulus, e.g., Young's modulus for uniaxial tension/compression, shear modulus for pure shear, and bulk modulus for volumetric deformations. It is important to emphasize that these definitions rely on strict assumptions that are rarely satisfied in soft biomaterials: they require the material to be isotropic and homogeneous, and the deformation to remain within the linear elastic regime. Experimental techniques commonly used to infer stiffness moduli in soft biomaterials, such as indentation, rely on mathematical relationships that also hold under the same stringent specifications. Outside these assumptions, metrics may enable comparisons between samples tested identically (e.g., at the same strain magnitude and rate), but they no longer represent intrinsic material parameters.

In its second meaning, stiffness refers to the response of a *structure* to prescribed deformation (e.g., axial stiffness of a bar or bending stiffness of a beam). Under realistic conditions, structures experience complex multiaxial deformation states where stiffness cannot be reduced to a single parameter. It also seems obvious that hardly any biological environment can be described in terms of bars and beams, although researchers have successfully modeled the cell's cytoskeleton using discrete structural elements like rods and cables [36].

Finally, stiffness is by definition a phenomenological parameter highly dependent on the scale of experimental assessment. For instance, fibrous soft tissues tested with classic experiments display stiffness metrics that are representative of the tissue scale. Testing a single fiber produces a different stiffness value because the averaging effect of the tissue architecture over the sample dimension is removed. A similar argument applies to porous soft tissues, where the tissue‐scale metric results from homogenizing the solid phase and the voids. Therefore, defining the scale of evaluation is essential for comparative analyses. Biomaterials with identical stiffness at the tissue‐level may exhibit completely different mechanical behavior at the cellular length scale.

### 2.1 Architecture of Soft Biomaterials

**FIGURE 3:**
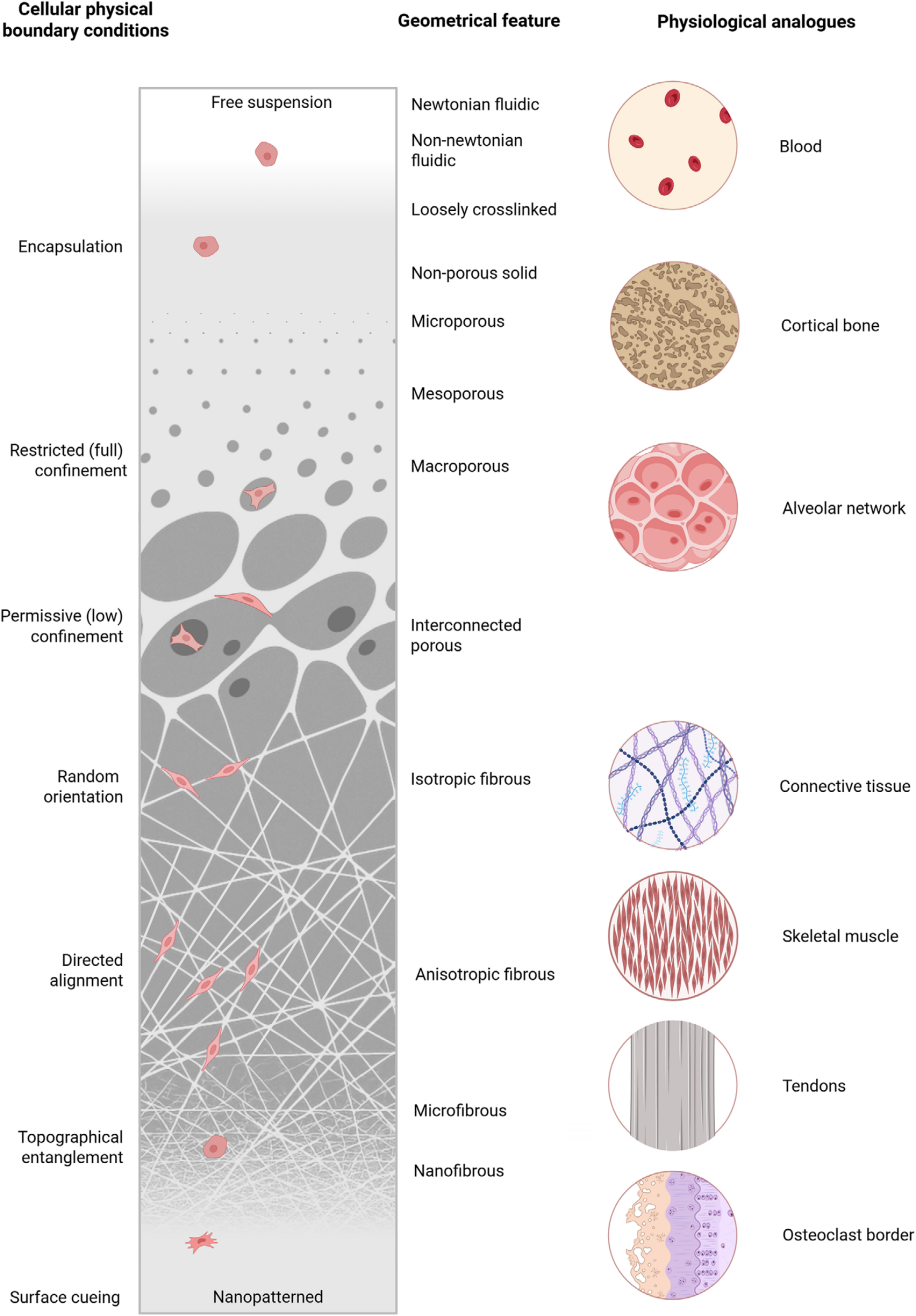
Schematic illustration of biomaterial architectures, their corresponding cellular interactions or restrictions, and physiological analogues from fluids to two‐dimensional topographical cues. From top to bottom, the materials progress gradually through different structural types. The depicted cell behaviors of attaching cells include encapsulation in single‐phase matrices, trapping within macro‐porous structures, attachment at throats – i.e., the interconnection of pores – or walls, and alignment or macroscopic orientation in fibrous scaffolds. This depiction highlights the influence of microstructure on cell‐material interactions, emphasizing design considerations for tissue engineering scaffolds (inspired by [58]). Please note that nanostructures are further distinguished and classified based on their pore size into microporous, mesoporous, and macroporous structures. These are established denominations that do not refer to the actual size in micrometers, but rather represent a standardized classification system [59].

In soft biomaterials, architecture describes the structural organization through which a material presents physical cues to cells. This includes not only pores, fibers, networks, and patterned surfaces, but also the absence of persistent structure in fluids or highly homogeneous matrices (see Figure 3). Establishing a clear terminology for these architectures is important because cellular responses in 3D biomaterial cultures are rarely determined by composition or stiffness alone. They also depend on how cells are spatially confined, where they can adhere, how forces are transmitted, and whether mechanical cues arise from the bulk matrix, local heterogeneity, or the cell‐material interface. A scale‐aware understanding of biomaterial architecture therefore provides a framework for interpreting experimental studies, comparing different culture systems, and distinguishing whether observed cellular behavior reflects material chemistry, mechanics, geometry, transport limitations, or their combined effects.

#### 2.1.1 Fluids Single‐Phase Materials and Nanoporous Gels

Fluids and single‐phase materials represent a non‐architected class of biomaterial environments. Unlike porous matrices, fibrous networks, or patterned substrates, they cannot be meaningfully categorized by architecture because they lack persistent structural organization at a cellular scale. Instead, they are primarily described by their composition, molecular content, viscosity, and physicochemical properties. Nevertheless, they exert a profound influence on cellular behavior through fluid resistance, hydrodynamic shear stresses, or pressure gradients [23, 37, 38, 39]. These environments provide passive mechanical feedback, such as osmotic pressure or geometric confinement, which can alter cellular motility even in the absence of direct adhesion [40]. Weakly or ‘loosely’ cross‐linked nanoporous architectures behave like fluids but create a special case of a mechanically responsive environment. In contrast to fluids, which lack structural integrity and do not support stable cell anchorage, these gels are able to provide sufficient mechanical cohesion to present anchoring points, enabling cells to exert traction forces, probe local stiffness, and initiate active mechanotransduction [41]. Because of their low cross‐linking density and high water content, some materials can behave as *ultra‐soft solids*—exhibiting both fluid‐like flow and solid‐like support [42]. They occupy a transitional regime between dispersions, non‐Newtonian fluids, and low‐viscosity solids. Consequently, the transition from passive (confinement‐ or flow‐mediated) to active (adhesion‐mediated) environments is determined less by the material's state alone (solid vs. fluid) and more by the presence of adhesive ligands, binding properties of monomers, polymers, or molecules, and size of dispersed particles with anchoring points. Importantly, polymers themselves, even in solutions or dispersions, can dynamically and (ir)reversibly adsorb and desorb at interfaces, thereby influencing biomechanical interactions.

These viscous, single‐phase or nanoporous systems offer a spatially uniform distribution of mechanical stress at the cellular level and highly tunable viscoelastic behavior on multiple scales, making them ideal substrates for controlled mechanotransduction studies in 2D cell culture systems [3, 43, 44]. In 3D, these materials function as matrices, either transient/degradable or stable, that physically confine cells while simultaneously promoting their viability [45, 46], cell proliferation, and tissue regeneration. Furthermore, the introduction of dispersed‐phase architectures—e.g., nanoparticles, microspheres, or fiber inclusions smaller than a single cell—further modulate these otherwise homogeneous mechanical environments by introducing stiffness gradients, damping effects, or stimuli‐responsive actuation [47, 48, 49].

#### 2.1.2 Structured Heterogeneous Materials

Unlike single‐phase or nanoporous systems, macro‐porous scaffolds—both bi‐phasic and multi‐phasic—lead to local micromechanical heterogeneity through the formation of discrete microenvironments within the material. These architectures are often inspired by tissues such as bone, lung alveoli, tendon, ligament, skeletal muscle, blood vessels, and the fibrous ECM of connective tissues, where oriented or interwoven fiber networks provide directional mechanical support and guide cell organization. They consist of pores, fibrous strands, and fiber families [50] (aligned or organized bundles of fibers that vary in orientation, density, and composition). These structural elements play a critical role in directing cellular alignment, force transmission, and matrix remodeling. Such local variations and heterogeneous elements at the microscale dictate how cells sense force, adhere, and migrate, providing a layer of complexity not found in single‐phase materials.

The inherent architecture of multi‐phasic materials facilitates multiple biological processes which are critical for tissue viability and cellular function [48, 50, 51, 52], including: mass transport, enabling perfusion and nutrient delivery; structural integration, supporting cell migration and active matrix remodeling; functional tuning, allowing for the independent adjustment of stiffness, degradability, and bioactivity. By integrating solid, fluid, or dispersed components, these systems maintain structural stability and biological adaptability [53, 54, 55, 56, 57]. The local micromechanical heterogeneity provides spatial cues necessary to drive complex behaviors like cell differentiation [44].

However, a significant challenge remains. While microscopic features directly influence cells' perception of their immediate surroundings, the biomaterials must also accurately match the bulk mechanical properties of the target tissue in order to reduce shear, friction, or pressure mismatches at the tissue interface. In dynamic culture systems or in vivo applications, materials must provide cell‐instructive micromechanics (e.g., local stiffness and viscoelasticity) without compromising macromechanical integrity (e.g., bulk elasticity). Optimizing a biomaterial at only one scale is often insufficient to meet both biological and structural requirements.

More recently, architecturally designed biomaterials have introduced an additional level of linking mechanical control to architecture. Unlike conventional multi‐phasic materials, these *meta‐biomaterials*—often fabricated via high‐precision additive manufacturing—exploit geometric features at the meso‐ or microscale. By utilizing structures such as auxetic lattices, re‐entrant honeycombs, or negative Poisson's ratio architectures, materials scientists can generate mechanical behaviors unattainable by conventional bulk materials [60, 61, 62, 63]. These architected geometries alter local strain distribution, deformation dynamics, and force propagation within the scaffold, thereby influencing cellular spreading, alignment, infiltration, and mechanotransductive signaling. In particular, auxetic structures with a negative Poisson's ratio can undergo lateral expansion under tension or densification under compression, dynamically modifying pore geometry and local mechanical cues experienced by cells during loading.

A key advantage of this approach is the ability to decouple micro‐ and macromechanical responses. By manipulating structural features alone, one can independently control typically linked parameters, such as stiffness and porosity, opening new capabilities for tissue integration and load‐bearing implants. Understanding these architecture‐dependent and scale‐dependent mechanical responses is therefore essential when analyzing multi‐phasic or structurally complex biomaterials, where the interplay between geometry and material composition governs cellular behavior and mechanical performance across multiple length scales.

#### 2.1.3 Nanotopography and Two‐Dimensional Architectures

Nanotopographical cues define the opposite boundary of architecture‐based biomaterial categorization. Because their features are smaller than the cell, they do not form a cell‐scale three‐dimensional microenvironment, but instead modulate the cell–material interface. Unlike nanoporous bulk materials, whose nanoscale pores contribute to a volumetric matrix surrounding or embedding cells, nanotopographical patterns act primarily as two‐dimensional architectures at the cellular scale. They guide adhesion, spreading, polarity, and mechanotransduction through local variations in feature size, spacing, curvature, and anisotropy [65].

Below the cellular length scale, architecture therefore no longer primarily defines the space available to the cell, but how the cell couples mechanically and biochemically to a material surface [50]. This coupling can influence focal adhesion formation, receptor clustering, membrane curvature, cytoskeletal organization, and cell polarity [65]. In this sense, nanotopography represents the terminal scale of architecture‐based classification, where material structure is sensed through subcellular contact geometry rather than through pore space or scaffold geometry.

Importantly, nanotopography is not restricted to flat substrates. It can also occur as a surface feature of larger micro‐ or macro‐level architectures, including porous scaffolds, fibers, lattice structures, and implant surfaces. In such systems, nanotopographical features add a subcellular interfacial layer to an already architectured material, allowing bulk geometry, microscale organization, and nanoscale contact cues to act together across length scales.

### 2.2 Mechanical Models of Soft Biomaterials

Mechanocompatibility of biomaterials is primarily linked to how materials respond to mechanical stimuli. Although such a response is embedded in the relationship between the input (e.g., force) and output (e.g., deformation) of a mechanical test, this relationship remains unknown until a *mechanical model* is formulated (Box 2). These models provide the link between the mechanical environment of a biomaterial and the biological responses by introducing measurable parameters that can be quantified, compared, and correlated with cellular outcomes. In addition, models become essential tools not only for material characterization and computational modeling but also for studying cellular behavior in deforming or active environments, and for extrapolating findings from in vitro scaffolds to larger biological systems.

**BOX 2 ‐ What is a Mechanical Model?**

Models are mathematical representations that describe the inherent behavior of a material as a function of external stimuli. In a *mechanical* model, these stimuli are of a mechanical nature. The construction of a mechanical model begins with a process of idealization in which only the aspects of mechanical behavior deemed most relevant to the problem at hand are retained. This step inherently entails a high degree of subjective decision‐making. It is critical to recognize that a selected model describes a material's response under specific conditions; the same material may require an entirely different model if environmental parameters (e.g., loading rate, temperature, humidity) are altered.

Given the relative nature of these descriptions, their usefulness may seem questionable. However, mechanical models provide the necessary framework to translate raw experimental data into material properties. Importantly, these parameters are not absolute descriptors of a material's mechanical response, but rather mathematical variables introduced by a specific model for the quantitative representation of material properties [66]. Therefore, experimentalists obtain phenomenological parameters that represent the mechanical behavior of a specific (bio)material only within the specific conditions of the test. However, a robust model extends beyond the laboratory, allowing researchers to simulate and predict behavior in the complex, multiaxial loading environments encountered in vivo. To achieve this, we need to ensure that the material parameters calibrated under the controlled laboratory environment undergo a validation step.

The following subsections outline the primary categories of modeling approaches used to describe the mechanical behavior of soft biomaterials and the related physiological analogues. Given the vast array of mathematical formulations in the literature, our objective is not to provide an exhaustive list, but rather a macro‐categorization, thereby establishing a range of possible model categories, within which specific models can be selected based on the intended application. To ensure the framework remains both practical and focused, our discussion is specifically restricted to formulations rooted in continuum mechanics, as this remains the most widely adopted framework in the analysis of soft biomaterials and tissues.

#### 2.2.1 Elasticity

The concept of *elasticity* originates from the work of Robert Hooke, who observed that in many solids the resulting displacement is proportional to the applied force (*ut tensio, sic vis*). In line with everyday experience, elasticity is commonly associated with the ability of a solid to return to its original configuration once the applied load is removed. More rigorously, a material is defined as elastic if: (i) its stress response depends solely on the deformation at that time instant and not on the deformation history; and (ii) the deformation process is reversible, such that all mechanical work is stored as strain energy, which is the form of potential energy of an elastic body. Strictly speaking, the second condition characterizes a subclass of elastic materials known as *hyperelastic*. However, in the literature the two terms are often used synonymously, therefore we do not further discuss the difference here.

Rather, it is crucial to recognize that the elasticity described by Hooke encompasses only a specific class of models, known as linear. Linear elastic models are used for describing local mechanical behavior at the cellular level [67, 68, 69], but are also sometimes erroneously used to characterize soft biological tissue [70, 71] and related biomaterials [72, 73, 74] at quasi‐static rates. Since linear elasticity provides an approximate description of material behavior, valid under a set of stringent mathematical assumptions, it is the responsibility of material scientists to verify whether these assumptions are met for a given experimental characterization. Beyond the linear regime, many solids behave elastically but show a nonlinear relationship between force and displacements (or stress and strain). In this case, the models describe a material's behavior without the mathematical restrictions of linear elasticity but involve more complex formulations and often a larger number of material parameters. Nonlinear elastic models are the most common choice for describing responses of soft tissues or scaffolds that undergo significant deformations [75].

#### 2.2.2 Viscoelasticity

Viscoelastic models are typically used to describe a material's response when rate‐dependent effects (i.e., relaxation, creep, hysteresis) are clearly identifiable in experimental characterization. In addition, it appears that viscoelasticity plays a critical role in mechanotransduction, as cells also actively sense the rate and duration of mechanical cues [76, 77, 78].

*Viscoelasticity* describes the mechanical response of a material that lies between that of an ideal elastic solid and a viscous fluid. From a thermodynamical point of view, an elastic solid is able to store all mechanical work performed during deformation as potential energy. In contrast, viscous fluids subjected to non‐hydrostatic stress cannot store energy, therefore exhibiting a purely dissipative behavior. A more practical definition is based on the material response to the sudden application of a uniform load (or deformation), which is then held constant. An elastic material responds instantaneously with a deformation (or stress) that remains constant over time.

A viscous fluid, on the other hand, undergoes continuous, steady flow. A viscoelastic solid exhibits a combination of both behaviors: an instantaneous deformation followed by time‐dependent flow known as creep (or, in the case of constant deformation, an instantaneous stress increase followed by stress relaxation). In classic mechanics, rheological models are used to schematize energy storage and dissipation associated with elastic and viscous responses, and different metrics are adopted to describe the property of a viscoelastic solid. When the material is exposed to oscillatory loading, a complex modulus is defined, which includes the elastic component of the response (the so‐called storage modulus) and the viscous, fluid‐like component (the so‐called loss modulus). It is important to emphasize that these concepts are rooted in a phenomenological approach to the mechanics of materials, without invoking or requiring any specific assumptions about the underlying microstructural mechanisms responsible for such behavior. For instance, in fibrous soft tissue stress relaxation and creep arise because of fiber uncrimping, inter‐fibrillar sliding, and gradual fiber alignment [79, 80], and spatially inhomogeneous architectures and/or deformations give rise to complex behaviors that challenge the direct applicability of simple viscoelastic models.

#### 2.2.3 Poroelasticity

In soft hydrated materials, rate‐dependent effects can arise not only from internal friction between constituents, but also from the redistribution of interstitial fluid in response to deformation, necessitating models that incorporate more than just the single‐phase solid. The simplest phenomenological theory describing the response of a material composed of a solid skeleton and an interstitial fluid is known as *poroelasticity*. In essence, it is a continuum theory grounded on a macroscale homogenization of porous solids, i.e., solids containing relevant fractions of connected void space. In addition to monophasic representations, poroelastic models incorporate mathematical descriptions of the mutual interaction between the solid matrix and the fluid phase. At an experimental level, they can describe the coupled response to solid deformation and fluid flow, manifested in phenomena such as consolidation and fluid‐driven compression. Common parameters, in addition to those pertaining to monophasic solid or fluid models, include porosity (the volume fraction of void space) and hydraulic permeability (the ease with which fluid flows through the pore network) [81].

By capturing interactions between fluid flow and deformation, poroelasticity is particularly relevant for cells or cell layers embedded within extracellular matrices or scaffolds, where fluid‐mediated stimuli—such as pressure gradients [82] or shear forces [83] (see also 5.2)—play fundamental roles in mechanosensing. These cues are particularly important under dynamic or cyclic loading, during which transient pressure and flow fields directly stimulate mechanosensors at the cell surface, like TRPV4 channels [84] or primary cilia [85]. Multi‐phasic models, which extend classical poroelasticity, also allow us to consider fundamental transport of solutes, nutrients, and metabolic waste, which are fundamental to characterizing cellular function under physiological conditions. In literature, poroelastic and multi‐phasic formulations have been applied to a wide range of soft tissues, most notably cartilage, intervertebral disc, skin, arterial tissue, and brain. More complex responses are obtained using a combination of models for solid materials (e.g., elastic or viscoelastic) with the multi‐phasic theories, resulting in poro‐viscoelastic formulations [86, 87, 88, 89].

#### 2.2.4 Poro‐Viscoelasticity

If the solid phase of a bi‐phasic or multi‐phasic assembly possesses intrinsic time‐ and rate‐dependent properties, the continuum is most accurately characterized as *poro‐viscoelastic*. Essentially, these formulations combine the poroelastic framework and related parameters—such as porosity and permeability—with a viscoelastic description of the mechanical response of the solid. Such models are increasingly used to describe the constitutive behavior of soft tissue characterizing cartilage [90, 91, 92], liver [93, 94], and brain tissue [95, 96], as well as their synthetic mimics [87, 88]. These models are particularly relevant at the cellular scale; because cell‐material interactions occur at the microscale, cells sense and respond to the localized, time‐dependent mechanics of surrounding matrix or fibers [76, 97]. Therefore, the poro‐viscoelastic model can more accurately bridge macroscopic mechanical responses with the microscopic mechanical cues that govern cellular function.

#### 2.2.5 Multiscale and Multiphysics Models

Cell size and the characteristic scale of biomaterial architecture both strongly influence cellular perception of mechanical cues, including stiffness, surface topography, and spatial confinement [98, 99, 100]. Micropatterned surfaces on the cell scale (∼10–100 µm) modulate cytoskeletal tension and signaling [101], while microrheology shows that matrices “feel” softer to cells at small scales than bulk stiffness suggests [102]. Nanoscale patterns further alter cellular adhesion by enhancing integrin turnover, mimicking softer environments despite rigid substrates [103]. However, structural and mechanical properties do not solely define the compatibility and mode of interaction of biomaterials with cells and living tissue. Equally important is the biological context in which the material is applied, which can significantly influence how forces are transmitted across cellular populations or extracellular constructs.

The mechanical environment of a biomaterial must be characterized across multiple scales, as it appears that both the macroscopic (tissue‐level) mechanical response and the microscopic (cellular‐level) cues ultimately determine its success. The continuum theory of mechanics offers a consistent framework to integrate relevant responses at different scales, preserving formulation clarity and efficiency. Specifically, we consider microstructure‐informed models and identify two macro‐categories: *implicit* (structure‐based) continuum models, which capture heterogeneous microstructural features and parameters indirectly, by integrating averaged fields within classic macroscopic constitutive laws. They are computationally efficient for global tissue‐level simulations but provide a homogenized view that may obscure localized mechanical events. The second category is that of *explicit* (micromechanical) multiscale models, which explicitly discretize the physical geometry and distribution of microconstituents, such as fibers or pore walls. These models typically utilize representative volume elements to describe the relevant microscopic features and assume this is the fundamental repeating unit of the material. Microscale behavior is upscaled to derive the macroscopic response through analytical or numerical homogenization. Notably, both approaches can integrate the general formulations described in the previous sections, although the actual implementation and solution might become non‐trivial [104]. Practical applications of these multiscale frameworks include direction‐dependent brain tissue response and the associated deformation at the cellular‐scale level in white matter [105], heart ventricular dilation relative to sarcomere number and myocyte length [106], and tumor cell migration within varying mechanical microenvironments [107].

While implicit models are computationally efficient for global tissue‐level simulations, only explicit frameworks can resolve local features, e.g., providing information about stress distribution within fibers. Their predictions can therefore be more directly translated to force transmission pathways that are otherwise lost in macroscopic continuum averages. Importantly, this multiscale concept can be repeated across different levels, where the smallest scale coincides with the highest level of resolution of structural and mechanical features. This way, the model can describe the fundamental features of a biomaterial's architecture (see Figure 3) and also the cell's size, both of which are known to influence cellular perception of mechanical cues [98, 99, 100]. In addition, through upscaling and homogenization, the information at the local level can be transferred to the macroscopic response, helping to provide a quantitative prediction of a biomaterial's behavior across scales [103].

The complexity of soft biomaterials often necessitates multiphysics models that couple mechanical behavior with additional physical or chemical stimuli. These frameworks can integrate: thermal effects, characterizing the temperature‐dependent response of biological tissues [108] and soft biomaterials [109]; biochemical influences, including pH, protein sensitivity, or enzymatic activity, modeling phenomena such as collective cell migration driven by extracellular signal‐regulated kinase (ERK) signaling [110]. In addition, while poroelastic models can provide insights on both solid‐ and fluid‐related fields, fluid‐structure interaction (FSI) models provide superior accuracy for modeling physical forces transmitted directly to cells, such as endothelial cells within a vessel [111].

In our perspective, these multiscale and multiphysics models represent the gold‐standard for addressing mechanocompatibility, since they can integrate architectural features and describe cellular responses to cues of different nature across scales. By resolving localized cues, such as gradients in stiffness (durotaxis), chemical concentrations (chemotaxis), or adhesive ligand patterns (haptotaxis), we can systematically investigate how cells generate force, adhere, and migrate within soft matter biomaterials.

## 3 Cell‐Material Interactions: How the Cell Perceives its Environment

While mechanical models describe the distribution and transmission of forces in biomaterials, their biological interpretation depends on interactions occurring across multiple hierarchical levels at the cell‐material interface. These levels include: (i) intrinsic material properties that define the local physicochemical environment, (ii) structural and geometric constraints that shape force transmission, (iii) cellular mechanisms of mechanosensory perception and adhesion that detect these cues, and (iv) emergent mechanical behaviors such as stress relaxation or nonlinear deformation that dynamically modulate cell responses over time. Although tightly interconnected, these categories represent distinct conceptual layers ranging from material design to cellular interpretation and ultimately to tissue mechanics.

Mechanical models can describe how forces are distributed and transmitted through biomaterials, but their detection and interpretation at the cellular level depend on cell‐material interactions. Put simply, the stiffness of a material can be a parameter to characterize its macroscopic mechanical response, but it is the local presentation of mechanical cues that determines how a cell perceives and responds to forces [112, 113, 114, 115]. For instance, protein‐mediated adhesion complexes at the cell‐material interface recognize not only biochemical ligands but also physical properties such as substrate flexibility, geometry, and nanoscale topography. These local cues ultimately drive cell behavior at the interface [116, 117].

Understanding these cell‐level mechanisms from the perspective of the cell is essential for designing biomaterials that are not only structurally and mechanically compatible with the tissue but also capable of effectively transmitting cues and guiding specific biological outcomes. Since many regenerative and therapeutic strategies rely on the precise control of cell behavior at the interface, dissecting how individual cells interpret and transmit mechanical information provides the foundation for engineering functional tissues and ensuring successful in vivo integration. Importantly, these categories should not be interpreted as isolated mechanisms, but as coupled layers of the mechanical microenvironment, where material properties define the available physical cues, structural organization governs force propagation, and cellular machinery determines biological interpretation and downstream response.

### 3.1 Material‐Level Interfacial Properties

The interfacial chemistry of biomaterials—such as surface charge, protein adsorption, hydrophilicity, and ionic content—can significantly influence cell behavior through passive physicochemical interactions [118, 119, 120, 121]. These factors operate independently of receptor‐mediated adhesion and play a crucial role in modulating protein adsorption, hydration layer dynamics, and even local osmotic gradients and immune responses [118, 121, 122].

Osmotic pressure changes at the interface—arising from leaching solutes from the material, ion exchange, or surface‐bound osmolytes—can subtly be sensed by the cell and modulate its mechanics [123]. For example, osmotic gradients across the membrane influence cytoskeletal contractility and nuclear deformation via mechanosensitive ion channels and tension‐sensitive transcription factors like YAP/TAZ [124].

Furthermore, the wettability of the material also affects protein adsorption, protein conformability, and integrin‐mediated adhesion. Hydrophilic surfaces expose fibronectin binding sites, thereby enhancing adhesion of the cell, whereas hydrophobic ones promote protein conformations that inhibit spreading, often leading to spheroid formation [125, 126, 127, 128]. According to the *Biomaterial Design Cycle*, shown in Figure 1, such properties can be systematically tuned via surface treatments like plasma oxidation, polymer brushes, or self‐assembled monolayers [129]. Surface charge alters the electrostatic landscape at the interface, influencing how ions and charged proteins distribute near the cell membrane [118]. Highly charged surfaces can recruit counterions and create Donnan‐type osmotic imbalances, thereby affecting the local cell volume, its membrane tension, and the vesicular trafficking. Cells exposed to charged interfaces often display altered spreading or membrane curvature, even in the absence of specific ligand‐receptor interactions [118].

### 3.2 Cellular Mechanosensation and Adhesion

At the cell‐material interface, mechanosensing arises from an integrated network of receptors and structures that physically couple ECM to the cytoskeleton and mediate cross‑talk between cells in vivo. Integrins are heterodimeric transmembrane receptors that link the ECM to the cytoskeleton and mediate cell‐matrix adhesion and mechanotransduction [130]. At adhesion sites, they transmit actomyosin‑generated contractile forces across the membrane through focal adhesion proteins [130]. Cadherins are calcium‑dependent adhesion molecules that transmit mechanical forces between adjacent cells and contribute to tissue morphogenesis [131]. Another important structure is the primary cilium, a small antenna‐like projection that detects mechanical cues such as shear forces caused by fluid flow and relays them into signals inside the cell. On the apical surface of endothelial cells, e.g., the pericellular glycocalyx senses fluid shear forces and conveys them to the cell by tethering to the cytoskeleton, regulating vascular permeability and signaling [132].

### 3.3 Structural and Geometric Constraints

**FIGURE 4:**
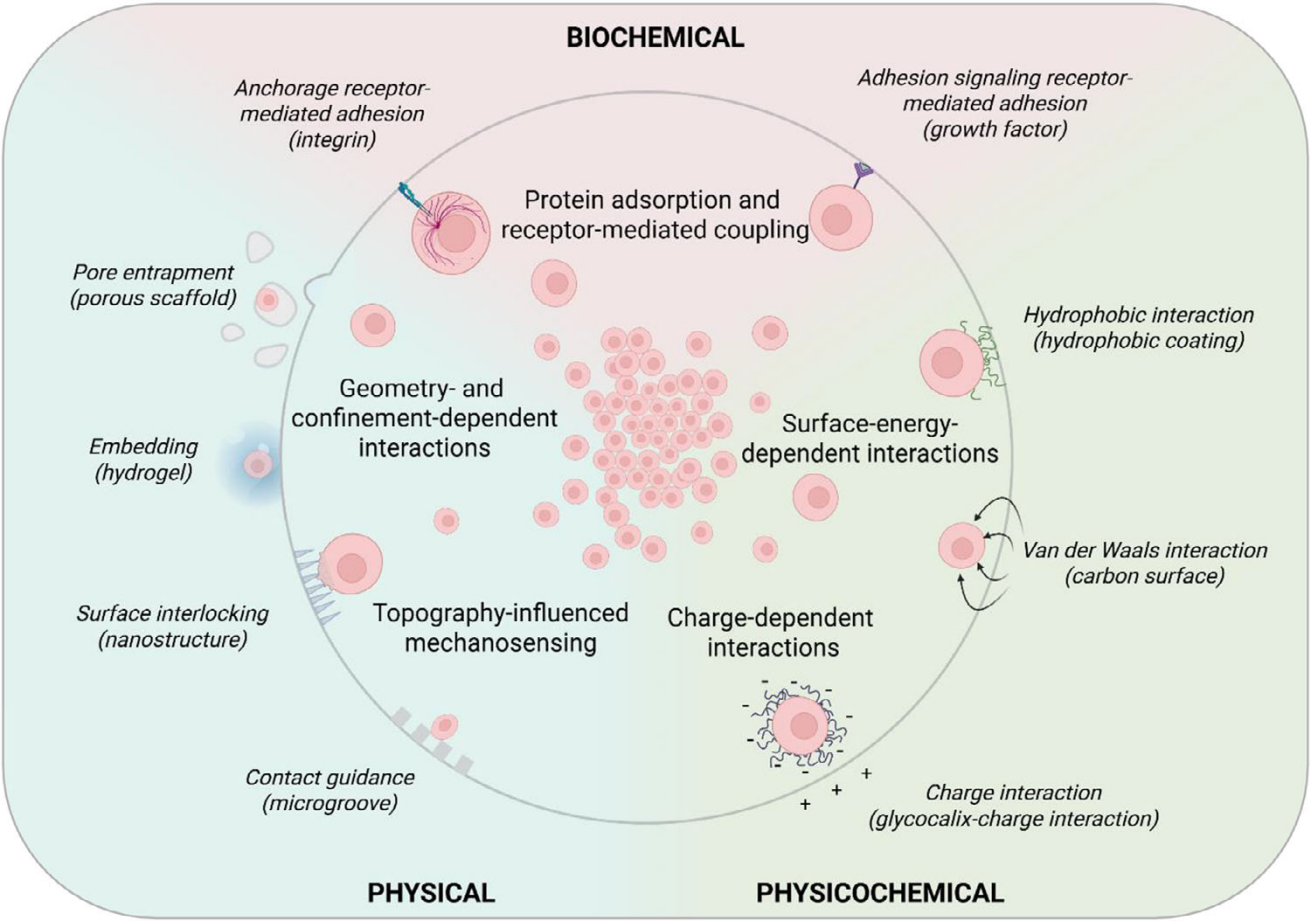
Schematic overview of the main mechanisms of cell‐material adhesion, showing dominant interaction modalities contributing to cell‐material coupling. The diagram illustrates three principal domains: physical, physicochemical, and biochemical adhesion, each with representative adhesion types and subtypes. The examples highlight how cells interact with material surfaces through geometric constraints, surface forces, or protein‐mediated processes. The mechanisms illustrated are not mutually exclusive but represent overlapping and dynamically coupled interaction modalities that collectively determine cellular attachment and biointerface formation.

In addition to the biochemical and physicochemical mechanisms discussed above, cells can also interact with materials through purely physical interactions that arise from confinement and matrix architecture. An overview of the dominant physical, physicochemical, and biochemical interaction modalities contributing to cell‐material coupling is illustrated in Figure 4. In viscoelastic encapsulations, soft 3D hydrogels or porous scaffolds, cells may become physically confined, with external geometry and pore architecture dictating force transmission [133]. Physical confinement acts as a geometric boundary condition that constrains cell shape, force distribution, and nuclear deformation, thereby influencing mechanosensitive signaling, cell fate, and maturation [134]. Confinement within stiff matrices, which promote higher cellular contractility, favors osteogenesis, while soft environments support adipogenesis [135]. In fibrous or microporous scaffolds, cells interact mechanically with matrix architecture, in some cases even in the absence of classical focal adhesions [133]. This mechanical interlocking enables cells to passively transmit forces via geometry rather than ligand‐binding and to activate mechanotransduction pathways [136].

## 4 From Biophysical Stimuli to Cellular Responses Across Scales

Mechanosensing at the single‐cell level lays the foundation for coordinated multicellular behaviors and ultimately drives tissue‐scale responses. To interpret these processes meaningfully, it is essential to review and understand the core principles of cellular mechanotransduction. The following section begins at the level of individual cells, where mechanical sensing initiates.

### 4.1 Single Cell Mechanosensitivity

Nearly all cells are mechanosensitive, yet the nature and magnitude of their responses vary with cell type. Immobile, anchorage‐dependent cells like fibroblasts form strong focal adhesions and stress fibers in response to stiffness, while motile or non‐adherent cells such as leukocytes respond transiently or through confinement‐based cues. Invasive and metastatic cells exhibit heightened mechanical adaptability, exploiting stiffness gradients for migration [137, 138, 139, 140].

In most cases, the cytoskeleton itself serves as a mechanosensor. Through actomyosin contractility and focal adhesion dynamics, cells regulate tension and respond to substrate stiffness [141]. The RhoA/ROCK pathway enhances actin stress fiber formation and contractility in stiff environments; conversely, soft substrates reduce RhoA activity, favoring motile or neurogenic phenotypes [142]. YAP/TAZ are key transcriptional regulators modulated by cytoskeletal tension. On stiff substrates, they translocate into the nucleus to activate growth and matrix‐related genes [141]. On softer substrates, they remain cytoplasmic, suppressing proliferation. This stiffness‐dependent localization plays a central role in development, regeneration, and pathologies like fibrosis and cancer [143].

Beyond cytoskeletal signaling that registers global changes in membrane tension, mechanosensitive ion channels provide another layer of mechanotransduction by rapidly modulating ion flux, membrane potential, and kinase activity. These channels allow cells to respond dynamically to small mechanical stimuli such as changes in curvature and minimal changes in membrane tension. PIEZO1 and PIEZO2 are primary mechanosensitive and non‐selective cation channels activated by membrane stretching (mainly PIEZO1), shear stress, and poking [144, 145, 146]. PIEZO1 is widely expressed in mechanically active tissues, whereas PIEZO2 is mainly present in primary sensory neurons and other specialized cell types [147, 148]. PIEZO1 is activated by the hydrophobic small molecule Yoda1 and the hydrophilic Jedi1 and Jedi2. For PIEZO2, currently, no activators are known. PIEZO channels mediate calcium influx, influencing processes such as vascular homeostasis [149], stem cell differentiation [150], phagocytosis [151], and apoptosis [152]. Through calcium signaling, they regulate cytoskeletal dynamics, mitochondrial activity, and cell fate, in part via ERK, MAPK, and NF‐κB pathways [145, 153, 154].

In addition to PIEZO channels, members of the transient receptor potential (TRP) channel family, also act as mechanosensors. These channels are sensitive to membrane stretch, osmotic swelling, and shear stress, and their activity is modulated by interactions with the actin cytoskeleton, lipid environment, and integrins [155, 156].

### 4.2 Multicellular Coordination and Emergent Tissue Mechanics

At the multicellular level, mechanical signals are transmitted not only through cell‐matrix adhesions but also propagated via cell‐cell junctions, enabling coordinated responses across tissues, which often increases the complexity of interpretation [157, 158]. In epithelial sheets, organoids, and dense 3D cultures, junctional tension and collective force generation support processes such as lumen formation, symmetry breaking, and directed migration [159, 160, 161]. These collective systems respond to confinement and external forces by self‐organizing into complex architectures and engaging in coordinated morphogenetic behavior [162, 163].

Cell‐cell interactions are mediated by molecules like cadherins (e.g., E‑cadherin), which form calcium‐dependent homophilic bonds linking the actin cytoskeleton for force transmission and coordinated morphogenesis [164, 165, 166]. In contrast, selectins (such as E‑ and P‑selectin) facilitate transient, shear‐sensitive binding via glycoprotein ligands, enabling leukocyte rolling and recruitment under flow conditions [167].

Cellular responses are shaped not only by local cues but also by emergent group‐level properties‐such as tissue stiffness, anisotropic stress, and curvature‐that feed back to influence gene expression, differentiation, and directional migration. Contractile cells generate long‐ranged stiffening gradients in their matrix, mechanically coupling neighboring cells over tissue scales [168, 169]. Such mechanical coupling enables cells to behave as integrated units in dynamic contexts like flow or cyclic loading. For example, epithelial tissues sense global curvature and use it to guide proliferation and alignment during branching morphogenesis [170, 171, 172]. At larger scales, these coordinated mechanosensitivities underlie processes like convergence‐extension, tissue folding, and wound closure. Understanding how signals are integrated from cells to tissues is thus crucial for translating in vitro mechanobiology into physiologically relevant settings.

Importantly, within such collectives, even cells with minimal or limited focal adhesions—due to sparse ligand presentation or inherent cell type differences—can still participate in mechanotransduction [18]. This is because mechanical coupling through adherens junctions or tight junctions allows cells to share and redistribute forces across the tissue. As a result, a single focal adhesion per cell may suffice for force transmission in tissue or in a mechanically integrated monolayer or tissue spheroid. This highlights the necessity of considering both cell‐cell and cell‐material interactions in dense cultures, where emergent mechanical behavior often overrides individual cell properties [173].

### 4.3 Organ‐Scale Mechanobiology

**FIGURE 5:**
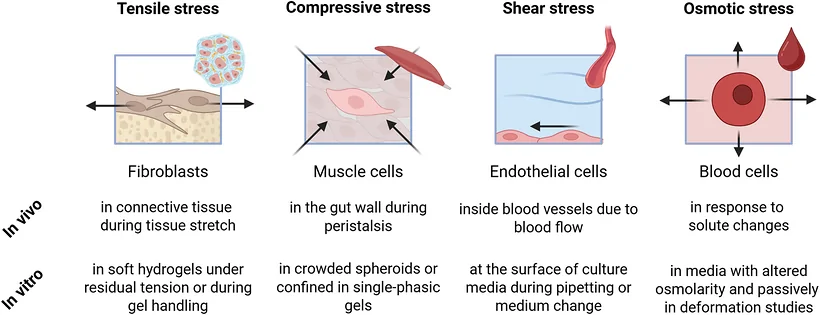
Schematic representation of various mechanical forces to which cells are exposed in vivo, with corresponding typical cell types and examples of in vitro occurrences. These forces can arise intentionally in vitro in mechanobiological studies or unintentionally due to residual tension, medium changes, unphysiological crowding, or altered osmolarity.

As tissues assemble into organs, new mechanical layers arise that are not present at the cellular or tissue scale alone. Organ‐level function introduces dynamic loading patterns, such as pulsatile flow in the brain or heart [174] and respiratory‐induced strain in lungs [175]. These stimuli are spatially and temporally heterogeneous, influencing not just local mechanosensing but also systemic coordination through neural or vascular interfaces. Moreover, organs often integrate multiple tissue types with distinct mechanical properties—such as muscle, connective tissue, and vasculature—which further complicates the simulation of complex mechanical processes in vitro. Mechanobiological feedback at this scale can drive emergent behaviors like perfusion‐dependent signaling, load‐induced remodeling, or compartment‐specific degeneration [143, 176]. To represent these effects in experimental models requires not only 3D architecture but also dynamic boundary conditions and multi‐tissue interactions. An overview of mechanical forces experienced by cells in vivo and their corresponding in vitro occurrences is shown in Figure 5.

At the organ scale, the Hippo signaling pathway integrates mechanical inputs with tissue growth, regeneration, and homeostasis [177]. This pathway converges on YAP/TAZ, whose nuclear translocation is suppressed when the Hippo kinase cascade (MST1/2‐LATS1/2) is active, thereby restricting proliferation and maintaining tissue size [178]. Mechanical cues such as ECM stiffness, cell density, and tissue stretch inhibit Hippo kinase activity, allowing YAP/TAZ to drive organ growth programs [177, 178]. In developing organs, YAP/TAZ ensure coordinated expansion of progenitor populations, while in adult tissues, they contribute to regeneration after injury by reactivating proliferation [177, 178]. Dysregulation of this mechanosensitive control promotes pathological tissue remodeling, fibrosis, and tumorigenesis. Thus, the Hippo pathway serves as a central mechanotransduction hub that scales cellular mechanical sensing into organ‐level growth control [177].

## 5 Biomechanical Stimuli in Tissue Engineering: State of Research and Cellular Relevance

Mechanobiology relies on experimental systems that replicate key aspects of the in vivo environment, such as tissue stiffness, geometry, environmental influences, and force exposure. These platforms—ranging from tunable substrates to microfluidic devices—enable controlled mechanical stimulation.

In native tissues, the mechanical environment is highly tissue‐specific, ranging from shear and tensile forces appearing in vessels to mainly compressive loading in cartilage and variable matrix stiffness across developmental stages [143, 179]. Reproducing these mechanical conditions in vitro is essential for the development of functional and physiologically relevant tissue constructs. Recent advancements in biomaterials and mechanobiology platforms now enable the control of such mechanical cues to guide cell fate decisions and promote tissue maturation [180]. The following section outlines major biomechanical stimuli, their native tissue contexts, and current in vitro strategies for their replication.

### 5.1 Substrate and Matrix Stiffness

The stiffness of the ECM is often described as a static mechanical cue. However, this description is a simplification: in living tissues, mechanical properties are rarely purely static. Instead, cells sense a combination of stiffness and time‐dependent stress‐strain behavior. This means that a matrix can feel soft in the short term but resist deformation over longer timescales, and cells integrate these signals when making fate decisions. For clarity, we will primarily refer to “stiffness” in the following sections as the metric provided by Young's modulus, bearing in mind the ambiguity of this concept as highlighted in Box 1, and point out that viscoelasticity constitutes an important nuance that refines rather than contradicts the general concept.

Matrix stiffness is sensed by cells continuously through cell‐adhesion molecules and cytoskeletal tension, profoundly influencing cellular behavior and fate decisions [181]. Physiological tissue stiffness spans a broad range, from soft neural tissue (with stiffness in the order of 1 kPa) to rigid bone (several tens of gigapascals (GPa), with cells responding through mechanotransduction pathways such as YAP/TAZ signaling and focal adhesion kinase (FAK) activation [2, 181, 182, 183].

In tissue engineering, tunable hydrogel systems are frequently used to control to direct stem cell differentiation. Soft matrices promote neurogenic and adipogenic lineages, while stiffer substrates enhance osteogenic orientation [181]. A widely used platform involves polyacrylamide hydrogels with adjustable stiffness from approximately 0.3 to 55 kPa, tuned via the acrylamide/bis‐acrylamide ratio, while maintaining a consistent chemical composition and surface functional groups suitable for cell culture [184, 185, 186].

Photocrosslinkable hydrogel systems further allow dynamic tuning of stiffness over time, facilitating studies on how temporal changes in mechanical cues affect development and disease progression. Studies show that mesenchymal stem cells (MSCs) cultured on stiffer substrates exhibit increased nuclear translocation of YAP/TAZ, correlating with enhanced osteogenic gene expression [187].

### 5.2 Shear Stress and Fluid Flow

Shear stress arises from tangential forces exerted by fluid flow on cell surfaces, as observed in interstitial fluid flow after tissue deformation or vascular environments, where blood flow generates defined shear patterns. For example, in vivo endothelial cells experience a range of wall shear stress within physiologic levels, from approximately 1 dyn/cm^2^ (0.1 Pa) in the vena cava and venules, to around 20 dyn/cm^2^ (2 Pa) in arterial vessels, and up to ∼50 dyn/cm^2^ (5 Pa) in small arterioles, depending on vessel type and geometry [188, 189, 190]. This mechanical stimulus activates mechanosensitive ion channels, promotes integrin‐mediated signaling, and induces cell alignment in the direction of flow [191, 192]. Beyond the cardiovascular system, renal tubular epithelial cells also experience shear forces generated by urine flow, influencing their structural organization and function [193], while osteocytes sense interstitial fluid flow‐induced shear stress during bone remodeling processes [194].

In 3D cell culture applications, vascular tissue engineering models various types of primary endothelial cells derived from different vascular beds, such as arterial, venous, or microvascular sources. These cells are cultured within microfluidic platforms to better replicate native vessel architecture and physiological shear stress environments [195, 196, 197, 198]. In kidney tubule‐on‐a‐chip models, proximal tubular epithelial cells exposed to shear stresses of ∼2–4 dyn/cm^2^ (0.2–0.4 Pa) display enhanced polarization, transport activity, and functional reabsorption capacity [199].

Commercial shear stress systems, such as parallel‐plate flow chambers driven by syringe or peristaltic pumps, can reproducibly generate physiologically relevant wall shear stress in vitro, typically ranging from approximately 0.01–30 dyn/cm^2^ (equivalent to 0.001–3 Pa), thereby matching the levels observed in vascular environments [200, 201]. These systems support various flow patterns, including continuous laminar, pulsatile, and oscillatory flow. Advanced microfluidic platforms further integrate multi‐channel designs for high‐throughput screening, with some systems enabling up to 929‐fold variation in local shear stress via adjustable channel constrictions [202, 203]. Notably, intermittent flow regimes that incorporate rest periods have been shown to significantly enhance osteogenic differentiation compared to continuous flow conditions [197].

### 5.3 Cyclic Stretch and Oscillatory Forces

Cyclic stretch simulates the rhythmic mechanical deformation experienced by cells in tissues such as the cardiovascular system, respiratory tract, and musculoskeletal structures. In vivo, cardiomyocytes undergo cyclic strain during each cardiac cycle, with physiological magnitudes typically around 10%–12% at normal heart rates [204, 205, 206]. Pulmonary epithelial cells similarly deform during breathing cycles, while vascular smooth muscle cells experience pulsatile stretch in response to blood pressure fluctuations. This mechanical stimulus activates stretch‐sensitive ion channels, induces cytoskeletal remodeling, and regulates gene expression programs associated with tissue development and maturation [205].

Cyclic deformation is typically implemented using vacuum‐actuated extension systems that deform silicone membranes, applying equibiaxial or uniaxial stretch to adherent cells. Controlled strain magnitudes of 10% relative to the original (unstretched) length at frequencies around 0.5 Hz are widely employed to replicate cardiac and vascular mechanics [206, 207, 208, 209]. In 3D tissue engineering, cyclic stretch is frequently applied to improve the structural and functional maturation of engineered cardiac tissues. These systems commonly use neonatal rat cardiomyocytes or human induced pluripotent stem cell‐derived cardiomyocytes embedded in collagen or fibrin‐based hydrogels [205]. In human cardiomyocyte cultures, physiological stretch promotes cell alignment, sarcomere formation, and upregulation of contractile proteins such as connexin‐43 and troponin T [206]. However, excessive strain (≥20%) has been associated with activation of inflammatory signaling cascade, such as Tribbles homolog 3 via TNF‐α and JNK pathways, as well as increased rates of apoptosis [210]. Transcriptomic analyses further demonstrate that cyclic stretch regulates pathways related to lipid metabolism, fibrosis, and structural remodeling, reinforcing the importance of applying physiologically relevant mechanical regimens [211].

### 5.4 Compressive Loads

Mechanical compression simulates the loading experienced in load‐bearing tissues, with articular hcartilage experiencing compressive stresses of 0.5–7.7 MPa during normal joint function [212, 213]. In vivo, chondrocytes subjected to dynamic compressive loading exhibit increased production of cartilage‐specific ECM molecules, most notably type II collagen and aggrecan. The magnitude, frequency, and duration of compressive loading are critical parameters, with dynamic compression generally proving more beneficial than static compression for promoting cartilage homeostasis and supporting chondrocyte‐mediated synthesis of ECM components [214].

In cartilage tissue engineering applications, human articular cartilage cells or MSCs are seeded in hydrogel scaffolds such as agarose or alginate to study chondrogenic differentiation under controlled compression [215, 216]. Pneumatically actuated compression systems are widely used to apply controlled cyclic loading to 3D hydrogel‐cell constructs. These systems enable precise and programmable application of compressive forces, typically up to several kilograms, to investigate the effects of dynamic mechanical stimulation on cellular behavior under reproducible conditions [214, 217, 218]. Multi‐well bioreactors have also been developed to combine compression and shear loading, simulating the complex mechanical environment of synovial joints [215]. Research demonstrates that cyclic compression enhances chondrogenic gene expression, with optimal protocols involving delayed application of compression after initial chondrogenic induction to maximize matrix synthesis while preventing hypertrophic differentiation [215].

### 5.5 Boundary Layer Effects

Since many tissues in vivo are naturally exposed to air or air circulation, such as the respiratory epithelium, skin, and other barrier‐forming epithelia, this exposure represents a physiologically relevant boundary condition [219, 220]. It imposes mechanical influences such as surface tension, stresses due to asymmetric hydration, and gradual spatial changes in physical or chemical conditions, all of which affect epithelial polarity, differentiation, and mechanosensitive signaling [221].

To replicate this in vitro, air‐liquid interface (ALI) cultures are used. An ALI culture exposes cells to air on the apical surface while maintaining basal contact with culture medium, thereby closely mimicking the native environment [222, 223, 224]. This setup supports cellular differentiation and organization in a direction that mirrors native tissue environments [220, 225]. Respiratory tissue engineering applications frequently use primary human bronchial epithelial cells cultured at ALI to generate pseudostratified epithelia with functional cilia and mucus production [219]. Commercial culture platforms such as Transwell (Corning Inc., Corning, NY, USA) inserts represent a widely used standard for air–liquid interface (ALI) models, as their porous membranes facilitate basolateral nutrient diffusion while maintaining apical air exposure, thereby enabling epithelial polarization and differentiation. More recently, gel‐ALI systems have been introduced, which integrate tunable‐stiffness hydrogels to better recapitulate the biomechanical properties of both healthy and fibrotic airway tissues [225]. Studies have shown that ALI cultures are critical for epithelial differentiation, as cells develop increased transepithelial electrical resistance and exhibit physiologically relevant responses to inhaled substances [220].

### 5.6 Rotational Forces and Suspension Models

In vivo, cells and tissues are exposed to several mechanistically distinct forms of rotational forces, each arising from specific geometric, fluid‐dynamic, or tissue‐mechanical conditions. Rotational flow patterns frequently arise at curved or branching vessel surfaces, where the geometry generates secondary vortices that impose rotating wall shear on adherent endothelial cells [226, 227]. Rotational forces can also originate within tissues themselves, for example, through the twisting motion of cardiac muscle, the helical deformation of connective tissue, or through rotational acceleration of the brain, each creating internal micro‐rotations and matrix‐transmitted shear strains [228]. A further category involves blood cells that are, in a physiological sense, suspended within the circulating flow, where erythrocytes and leukocytes experience tank‐treading, tumbling, and vortical shear while passing through disturbed or swirling regions of the bloodstream [229, 230]. In addition, rotational behaviors can emerge at the tissue‐aggregate scale, although this primarily becomes relevant in vitro: multicellular aggregates such as organoids undergo slow spinning in rotating‐wall environments [231, 232], while epithelial tissues cultured on curved substrates exhibit coordinated, curvature‐guided migration that minimizes local shear stress [233, 234, 235]. Surface‐tension‐driven epithelial budding and rotation in 3D tissues have further been demonstrated in organoid systems.

To investigate these different rotational patterns in vitro, and to recreate them at the level of 3D cell culture, a spectrum of specialized systems is used. Curved microfluidic architectures mimic interfacial vortices [236], viscoelastic 3D matrices reproduce internal, strain‐driven micro‐rotations, stirred suspension bioreactors generate vortex‐rich shear acting on blood cells and enable scalable culture [237, 238, 239], and spinning or rotating‐wall bioreactors impose controlled low‐shear rotation of multicellular aggregates and organoids, supporting uniform spheroid formation and microgravity‐like dynamics [231, 232, 240, 241]. Together, these complementary platforms allow both the systematic study and the targeted reproduction of the diverse rotational environments that shape cellular behavior in vivo.

### 5.7 Electrical Stimulation in Mechanobiology

The generation of electric potentials by mechanical deformation, known as the piezoelectric effect, is a fundamental biophysical property observed in mineralized tissues as well as cellular components such as collagen and cytoskeletal proteins [242, 243]. While whole cells are not piezoelectric in the strict physical sense, they do respond to mechanical forces with changes in membrane potential and ion flux, largely mediated by mechanosensitive channels and electrophysiological signaling [244, 245, 246]. These bioelectric responses differ from the lattice‐level piezoelectricity of structural biomolecules but illustrate how mechanical stimuli are intrinsically coupled to electrical cues in biological systems. This duality provides a biological precedent for designing in vitro platforms that emulate stretch‐induced fields as part of mechanobiological stimulation. To this end, piezoelectric scaffolds have been integrated into stretch chambers, compression bioreactors, and ultrasound‐responsive culture systems, enabling simultaneous application of mechanical loading and localized electrical cues [242]. For example, fibroblasts cultured on poly‐L‐lactic acid (PLLA) piezoelectric nanofiber scaffolds exposed to cyclic stretching or low‐frequency ultrasound generate bioelectric surface potentials that promote proliferation, collagen deposition, and directed migration, thereby replicating aspects of tissue remodeling under mechanical load in vitro [247, 248, 249, 250].

### 5.8 Topographical Cues

Previously discussed loading conditions can be induced and controlled by the structural design of the biomaterials. In native tissues, topographical cues arise from microscale and nanoscale structures within the extracellular matrix, cell surfaces, and tissue interfaces, guiding cellular behavior during development, healing, and homeostasis. These physical features influence cell adhesion, migration, and differentiation by interacting with cellular protrusions and organizing focal adhesions. In tissue engineering, micropatterned surfaces leverage such cues to control stem cell fate, enabling lineage‐specific differentiation (e.g., osteogenic, neurogenic, myogenic) even without soluble biochemical factors [116, 251].

Fabrication techniques such as photolithography enable the creation of precise micropatterns, while soft lithography allows the replication of complex surface geometries. Electrospinning is widely used to produce fibrous scaffolds that mimic the structure of the native extracellular matrix. Emerging additive manufacturing approaches, including two‐photon polymerization 3D printing, offer sub‐micrometer resolution and the ability to spatially control both cell placement and local mechanical properties within complex architectures [252]. Studies show that aligned fibrous scaffolds promote tendon‐like tissue formation, whereas randomly oriented fibers enhance osteogenic differentiation via mechanotransduction pathways such as YAP/TAZ signaling [253, 254, 255]. The integration of these physical cues within advanced bioreactor systems facilitates the generation of physiologically relevant tissue models, improving the replication of native tissue function and architecture in vitro [256].

## 6 Experimental Strategies for Assessing Mechanobiological Responses

Mechanotransduction cannot be deduced from a single endpoint assay, as mechanical information is transformed at multiple biological levels, from force application at the cell‐material interface to molecular, cytoskeletal, nuclear, transcriptional, proteomic, and functional adaptations at tissue‐level. Consequently, mechanical responses should be assessed using complementary assays selected based on the specific phase of mechanotransduction they interrogate, the biological scale they resolve, and the type of mechanobiological information they provide. In this context, the key consideration is not simply which technique is applied, but whether it captures signal initiation, force transmission at the cytoskeletal level, nuclear integration, or downstream functional mechanical output.

Signal initiation at the cell‐material interface can be most directly captured by image‐based assays. Immunofluorescence is particularly useful when the relevant question is whether a mechanical cue changes the localization of a mechanosensitive effector, such as YAP/TAZ nuclear accumulation on stiff matrices [257, 258, 259]. Confocal microscopy extends this by resolving 3D cytoskeletal and cell junction remodeling, while Total Internal Reflection Fluorescence (TIRF) provides selective access to the basal membrane and is therefore especially informative for investigating newly formed focal adhesions and their turnover [260, 261, 262]. Super‐resolution approaches provide significantly more information by resolving the nanoscale organization of the mechanotransduction machinery itself; in particular, iPALM showed that integrins, adaptor proteins, and actin form vertically stratified layers within focal adhesions [261, 263]. Live‐cell imaging adds the temporal dimension required to distinguish transient deformations from adaptive remodeling processes, such as strain‐rate‐dependent realignment of stress‐fibers and MAPK activation under cyclic stretch [264]. FRET‐based (Förster Resonance Energy Transfer) tension sensors move one step further by measuring picoNewton loads across specific proteins, thereby revealing not only the localization of a molecule, but also its mechanical load, as demonstrated for vinculin [265, 266, 267, 268]. In contrast, TEM and SEM mainly serve as confirmatory ultrastructural tools; they can validate the organization of focal adhesions and F‐actin organization or mechanically induced morphological changes, but cannot resolve dynamic signaling pathways and should therefore be considered as supporting rather than primary mechanotransduction assays [269].

Nuclear integration and gene regulation require assays that go beyond mere localization and investigate whether mechanical input has altered the signal state, transcription, or chromatin. While Western blotting is suitable for confirming changes in phosphorylation or the amount of pathway components, its averaging makes it insensitive to spatial heterogeneity, and it should not be overinterpreted as the sole method for mechanistic measurement [257]. ELISA is even further downstream but can be valuable for investigating the translational question of secreted inflammatory or pro‐regenerative mediators that are altered by stiffness or deformation [270]. At the transcriptional level, quantitative real‐time PCR (qPCR) remains the most efficient way for validating predefined, mechanoresponsive target genes, while bulk RNA‐seq is more powerful for detecting stiffness‐sensitive programs without prior bias [141, 271, 272]. Single‐cell RNA‐Seq (scRNA‐seq) is especially informative when the system has responder and non‐responder subpopulations or a diversification of pathological conditions, as it reveals heterogeneities that remain masked in bulk measurements. Spatial transcriptomics adds a tissue dimension by linking gene expression programs to local mechanical niches, with its mechanobiological interpretability being greatest when combined with stiffness or tension maps [273]. Finally, ATAC‐seq, ChIP‐seq, and CUT&Tag address the regulatory layer of mechanotransduction. ATAC‐seq identifies chromatin opening and mechanical priming, ChIP‐seq can reveal direct YAP/TEAD or AP‐1 occupancy at enhancers, and CUT&Tag offers a method with lower sample requirements for investigating similar questions in small sample sizes [274, 275]. These assays are particularly important when the translational claim relies on the detection of durable reprogramming rather than acute pathway activation.

Functional force output should be treated as a distinct endpoint and not indirectly derived from morphology or signaling. Traction force microscopy (TFM) is the most established method for determining whether altered signaling leads to altered cell‐generated forces. Importantly, multidimensional TFM has shown that focal adhesions during migration support not only shear tractions but also normal forces and rotational moments [276, 277]. Atomic Force Microscopy (AFM) addresses a different but complementary aspect of the response by quantifying apparent stiffness, viscoelasticity, adhesion, and nuclear mechanics. In stem cell systems, AFM has shown that mechanically distinct cell states are associated with differentiation and that differentiation is linked to altered nuclear architecture and mechanics [278, 279, 280, 281, 282]. Optical tweezers fill a complementary niche, as they can apply calibrated local loads and can probe receptor recruitment, membrane tension, or cortical viscoelasticity at the subcellular level. They have been used both to trigger the recruitment of focal adhesion under controlled force and to quantify the rheology of fibroblasts, neurons, and astrocytes [283, 284, 285, 286, 287]. Taken together, these assays determine whether a candidate material changes not only signal markers but also mechanical function.

*Multimodal combinations* offer the strongest basis for translational interpretation, as they close inferential gaps across different biological scales and link early mechanosensory events with durable biological outcomes [288]. Due to their hybrid functionality, some techniques cannot be assigned to a single methodological category: FRET‐based tension sensors combine molecular engineering with fluorescence imaging to capture picoNewton‐scale forces in real time, thus linking protein localization with direct load measurements on cell‐matrix and cell‐cell adhesions [265, 268, 287]. TFM, on the other hand, transforms imaging of bead displacements in compliant substrates into spatially resolved maps of cell‐generated traction [289]. Although TEM and SEM primarily perform structural investigations and are traditionally classified as image‐based techniques, they also provide corroborative ultrastructural evidence for mechanically induced remodeling processes and enhance the interpretation of mechanoresponses [290, 291]. When combined with localization imaging and transcriptomic or epigenomic profiling, these cross‐category approaches enable molecular loading, cellular traction, and chromatin‐level adaptation to be interpreted within a single mechanobiological framework. Recent studies that explicitly link molecular tension with traction measurements, as well as paired chromatin‐transcriptome analyses, further underscore the value of this integrative strategy [288].

## 7 Evaluating the Translatability of In Vitro Findings to In Vivo Applications

In order to ensure a comprehensive and integrative analysis as a basis for evaluating the translational potential of state‐of‐the‐art methods in mechanobiological studies, a structured approach was chosen.

### 7.1 Methodological Framework

The selection criteria prioritized current research on effect‐ or scale‐based hierarchical frameworks. Studies with experimental approaches, computational modeling, or biomedical engineering applications were included to ensure a multidisciplinary approach. Studies lacking general in vivo validation, and those without a fundamental explanation of results, were excluded. Only soft matter systems were considered—including natural ECM‐based matrices, naturally derived polymers, and synthetic polymers. Materials relevant to hard tissues, such as compact cortical bone, were included only for contextualization and to provide a comprehensive picture. Filtering by specific cell, tissue, or organ types was not performed due to the risk of excluding relevant information. However, particular attention was paid to studies with adherent cells. All results are considered, where appropriate, in the context of cell type or cell structure and architecture. Disease models and models with additives that alter the mechanosensitive response of cells—e.g., Mannitol acts as an osmoticum and alters the cellular response to increased osmotic pressure—were excluded to focus on fundamental mechanobiological responses and are only mentioned when relevant to the context [292].

Once a common language and uniform classifications were established, a thematic synthesis approach was applied to identify and interpret patterns across different studies and to integrate the results into coherent themes. Mechanistic insights from in vitro experiments were considered in the context of tissue‐ and organ‐level responses to link biomechanical phenomena at the micro and macro levels. To assess translational relevance, recent peer‐reviewed articles illuminating mechanistic pathways across multiple experimental scales, including monoculture, 3D cultures, organoids, and in vivo–adjacent systems, were compiled. The selection criteria focused on cellular responses to mechanical stimuli and excluded disease‐specific models. Particular attention was paid to experimental design, mechanotransduction pathways, and system complexity.

The translatability of in vitro studies to mechanobiology requires a balance between experimental control and physiological relevance. While simplified culture systems provide mechanistic insights, their predictive value for in vivo results depends on how accurately they reproduce native biophysical cues and cellular contexts. To systematically assess this, we compare studies across different levels of biological and structural complexity as described in the methods section, highlighting their strengths, limitations, and translational potential. The following sections introduce a structured framework for context‐based analysis, identify correlations between model complexity and outcome relevance, discuss the inherent limitations of in vitro assays, and highlight validated mechanobiological findings that successfully bridge the gap to in vivo physiology.

### 7.2 The Mechanobiological Fidelity Score

To formalize insights, we propose a *Mechanobiological Fidelity Score (MFS)* that scores a given model along four axes: mechanical representativeness, cell source, cellular organization, and timescale—each on a 0–2 scale. The sum yields a score from 0 (low mechanobiological fidelity) to 8 (high mechanobiological fidelity). For instance, a short‐term culture of immortalized fibroblasts without physiologically accurate stimulation scores only 1/8, whereas a microfluidic shear system in a synthetic 3D matrix with primary endothelial cells stimulated over a time period of 3 days scores 7/8, and a dynamic biomimetic 3D hydrogel supporting organoid growth with stromal/immune co‐culture under cyclic loading for 120 days reaches the maximum 8/8. This framework does not replace biological nuance but offers a comparative rubric for evaluating mechanobiology models and identifying where improvements—be it in input fidelity, context, or experimental duration—would most increase predictive value.

The MFS is introduced here as a comparative heuristic for assessing mechanobiological fidelity, not as a validated predictor of transferability to in vivo or clinical applications. Its purpose is to structure the comparison of in vitro mechanobiology models by evaluating how comprehensively a given system reproduces important context‐relevant dimensions, namely mechanical representativeness, cell source, cellular organization, and timescale. This distinction is important because predictive validity is domain‐specific and, for advanced in vitro systems, typically requires external benchmarking based on qualified in vivo results in a clearly defined application context. An overview of score criteria can be found in Table 1, whereas the final score assessment is summarized in Table 2.

**TABLE 1:** Overview of score criteria.

	Definition	Score 0	Score 1	Score 2
Mechanical representativeness	Degree to which mechanical aspects mimic in vivo conditions (stiffness, flow, cyclic strain, viscoelasticity)	Non‐physiological or static inputs	One key mechanical parameter matches physiology (e.g., shear or stiffness)	Multiple dynamic, physiologically relevant mechanical cues are reproduced
Cell Source	Relevance of cell type and retention of native mechanosensitivity	Immortalized cell line	Primary cells from related tissue or mixed co‐culture	Tissue‐derived/patient‐derived cells or target‐tissue primary co‐culture
Cellular Organization	Biological microenvironment fidelity (ECM & cell–cell interactions)	Simple monoculture; little to no cell–cell interaction	Basic co‐culture or native ECM inclusion	Multicellular system with stromal/immune/vascular niche components
Timescale	Ability to capture mechanoadaptation and remodeling	Acute exposure only (< 24 h)	Intermediate timescale (multi‐day adaptation)	Long‐term (>1 week) enabling remodeling/cyclic conditioning

**TABLE 2:** Final score assessment.

Total Score	Interpretation	Description
0–2	Low Mechanobiological Fidelity Score	Oversimplified; findings may lack physiological relevance
3–5	Moderate Mechanobiological Fidelity Score	Some translational value, but missing key biomechanical cues
6–8	High Mechanobiological Fidelity Score	Robust biomechanical validity; suitable for preclinical modeling

Table 3 summarizes and comparatively evaluates selected representative studies that have made significant conceptual, methodological, or translational contributions to the field of cellular mechanobiology.

**TABLE 3:** Main results of the individual studies, systematically categorized by defined criteria (key in vitro findings, mechanical/structural stimulus, physiological accuracy of the stimulus, cell and culture type, and cellular organization). The table also includes the Mechanobiological Fidelity Score (MFS) introduced as a measure of translatability. Scores of “0” are highlighted in light peach.

SINGLE CELL EXPERIMENTS							
Summary and Key Finding	Evaluation Method	Stimulus	Mechanical Representativeness	Cell Source	Cellular Organization	Time Scale	MFS
Mouse hippocampal neurons cultured on stiff polydimethylsiloxane (PDMS) substrates showed enhanced calcium channel activity, stronger Ca^2^^+^ oscillations, and increased excitatory synaptic connectivity and transmission compared to soft substrates; it is shown that substrate stiffness is a key biophysical cue regulating neuronal network activity in vitro [295].	Whole‐cell patch‐clamp recordings, calcium imaging, and immunofluorescence/confocal microscopy were used to assess cellular responses; substrate stiffness was evaluated using AFM	Substrate stiffness	0: Used static stiffness as only mechanical cue; no viscoelasticity, no dynamic loading, stiffness (46 ± 11 kPa and 457 ± 39 kPa) does not match the range of brain tissue stiffness	1: Primary mouse hippocampal neurons cultured from neonatal brain	1: Non‐confluent monoculture on single‐phasic substrate with cell‐cell interactions; some glial contamination is mentioned, but no intentional co‐culture or stromal context	2: 14–18 days	4
Stiffer coatings increase proliferation and promote YAP nuclear localization [296].	Immunofluorescence staining was used to assess YAP nuclear localization	Substrate stiffness	1: Uses hydrogels of varying stiffness (0.5, 4, 25 kPa). Substrate conditions are reasonably biologically relevant	0: SKOV‐3 cell line (ovarian cancer cell line, immortalized)	1: Monolayer culture on coated substrate	1: 3–6 days for proliferation	3
Cells on softer gels exhibited significantly lower Young's modulus and reduced adhesion forces, suggesting reduced cytoskeletal tension and focal adhesion formation; this mechanical feedback influenced cell spreading, actin organization, and migration behavior; fibroblasts adapt their internal stiffness to match the stiffness of their substrate [297].	Single‐cell AFM was used to assess cell stiffness and adhesion forces across substrates of differing stiffness; actin cytoskeleton organization was assessed by phalloidin staining and Western blot for F‐actin crosslinking	Substrate stiffness	2: Substrate stiffnesses chosen (PA gels 0.7–20 kPa) match soft tissue range (brain ∼0.1 kPa, fat 100 Pa) up to stiff glass; controlled fibronectin coating	0: NIH‐3T3 fibroblasts (immortalized mouse cell line)	1: Monolayer on gel substrates with possible cell‐cell interactions	0: Acute responses measured only (24 h)	3
Live‐cell fluorescence imaging during isotropic compression via light‐activated microgels reveals rapid, reversible intracellular calcium signaling [298].	Live‐cell fluorescence imaging during isotropic compression	Compression	2: Mechanical stiffness is tunable, compression is isotropic and physiologically relevant (forces up to 400 nN, strain up to 15%); this is a very close mimic of real tissue mechanics	0: Murine MSCs (ATCC D1 line) are used, an established murine cell line	1: 3D encapsulation of single cells within a tunable, cell‐adhesion‐modified alginate matrix, with high spatial control and optically actuated compression; absent cell‐cell interactions	1: Up to 1 week	4
In a rat CDH model, fetal cardiomyocytes from hypoplastic hearts show reduced (trend‐level) proliferation and immaturity in vivo but become more proliferative yet still immature in 2D culture; ECM from CDH+ hearts (solubilized and coated) suppresses proliferation of both healthy and CDH+ cells compared to healthy ECM, while cyclic stretch (1 Hz, 5%) decreases proliferation in CDH+ cells but enhances their sarcomere maturation without affecting healthy cells [299].	Mechanosensitive responses assessed via Ki67 staining, α‐actinin immunofluorescence, qPCR, and sarcomere length quantification	Cyclic stretch	1: Cyclic mechanical stretch in phsyiological relevant range (1 Hz, 5%); culture	2: Fetal cardiomyocytes from a rat model (native cells)	1: Cells are isolated and cultured in 2D on a deformable membrane	1: Static culture up to 4 days, cyclic stretch for 3 days	5
Exposure to hyperosmotic pressure (320→380→460 mOsm/kg, PEG or sorbitol) induces distinct single‐cell subpopulations with prolonged or arrested cell cycles, impaired nuclear growth and slowed migration; these states (measured by FUCCI2 time‐lapse, nuclear‐area quantification, single‐cell tracking and p21/Ki67 IF) are reversible after mild stress (380 mOsm) but persist after high stress (460 mOsm) (experiments: monolayer MDA‐MB‐231/MCF7 FUCCI2 cells on glass‐bottom microplates; 90 h imaging; reactivation follow‐up to 180 h) [300].	Real‐time quantitative time‐lapse imaging of cell cycle phases at the single‐cell level with FUCCI2 fluorescent reporter system. Immunofluorescence staining for p21 (cell cycle arrest marker) and Ki67 (proliferation marker).Cell migration assessed via single‐cell tracking; nuclear growth quantified by area measurements	Hyperosmotic stress	1: Substrate osmotic pressure was modulated in biologically relevant ranges (320–460 mOsm/kg) using accepted osmolytes	0: Immortalized human breast cancer cell lines, MDA‐MB‐231 (mutant p53, highly metastatic) and MCF7	1: Subconfluent monolayer culture in petri dishes	1: Tracking started after 24 h, the following osmotic pressure experiments spanned 90 and reactivation tests 180 h	3

MONOLAYER EXPERIMENTS							
Summary and Key Finding	Evaluation Method	Stimulus	Mechanical Representativeness	Cell Source	Cellular Organization	Time Scale	MFS
Matrix elasticity alone directs lineage choice in sparse mesenchymal stem cells (MSC) monocultures; soft gels (brain‐like) induce neurogenic markers, intermediate stiffness (muscle‐like) induces myogenic markers, and stiff matrices (osteoid‐like) induce osteogenic markers within ∼1 week; active non‐muscle myosin II contractility is required, and after ∼3 weeks, the matrix‐defined lineage becomes irreversible [44].	Immunofluorescence for lineage markers (β3‐tubulin, MyoD1, CBFα1), Western blotting, microarray transcript profiling, AFM indentation for substrate stiffness and cell mechanics, paxillin immunostaining for focal adhesions, and traction mapping and cell‐prestress quantification	Substrate stiffness	1: The magnitudes were chosen to mimic native tissue stiffness, so the values are physiologically relevant; however, the system is purely elastic. Stiffness included: ∼0.1–1 kPa (brain‐like), ∼8–17 kPa (muscle‐like), ∼25–40 kPa (osteoid‐like)	2: Primary, naive MSCs isolated from bone marrow	0: Low‐density monoculture of MSCs with no cell clusters.	1: 7 days (core assays); some 1 week + 1 week reprogramming tests; "commitment" by ∼3 weeks	4
Laminar shear stress induces endothelial cell elongation and alignment through active microtubule remodeling and increased microtubule acetylation; genetic or pharmacological perturbation of microtubule acetylation (HDAC6 and αTAT1) blocks elongation and alignment; microtubule acetylation is shown in vitro and in vivo with spatial flow relevance [301].	Immunofluorescence for cytoskeletal components, live cell microscopy, CRISPR and pharmacological manipulations, AI‐based Image segmentation, Western blotting	Laminar shear stress	1: Physiologically relevant shear stress magnitude (∼20 dynes/cm^2^), well‐controlled flow conditions	2: Primary human endothelial cells (HUVECs, HAECs)	1: Confluent monolayer culture	1: Responses were assessed 24–48 h after flow initiation	
Increased substrate stiffness promotes inflammatory phenotype in primary human BMECs; exposure to modest fluid shear stress (1.7 dyn/cm^2^) partially attenuates inflammation, including reduced chemoattractants and ICAM‐1 levels [302].	Molecular profiling (mRNA, protein markers), soluble factor quantification	Substrate stiffness and shear stress	2: Shear (1.7 dyn/cm^2^) and stiffness (6 vs 30 kPa) in physiological range	2: Primary human BMECs	1: 2D gelatin‐coated, flow‐conditioned monolayer culture	1: Cells adhered ∼24 h, then were put under flow for 48 h	6
Chondrocytes show robust transcriptional responses to 20% cyclic stretch (1 Hz, 2 h), including 29 stretch‐responsive genes linked to cartilage homeostasis and disease [205].	RNA‐mediated oligonucleotide Annealing, Selection, and Ligation (RASL‐seq), gene expression profiling, cell death imaging (EthD‐1), live cell counts, FEM and DIC strain validation	Cyclic stretch	1: Stretch magnitude/frequency physiologically relevant (20%, 1 Hz, 2 h) and validated by DIC/FEM; the silicone elastomer membrane was not validated for mechanical properties	2: Primary human chondrocytes	1: 2D monolayer in collagen‐coated silicone wells, high‐throughput compatible	0: Short‐term (2 h)	4
Cyclic stretch (10%, 1 Hz, 48 h) of hiPSC‐derived cardiomyocytes increases alignment, sarcomere length, gene expression for cardiac maturation, nuclear volume, LINC complex, Lamin A/C, YAP nuclear translocation, and promotes functional maturation via nuclear mechanotransduction [208].	Immunofluorescence (sarcomere, F‐actin, YAP, LINC/lamin), confocal microscopy, qPCR (maturation, YAP/TAZ/LINC genes), beating analysis, nuclear morphometry	Cyclic stretch	1: Cyclic stretch in the range of physiological cardiomyocyte cyclic stretch magnitude and frequency (10% strain, 1 Hz,); PDMS (10:1 Sylgard 184) is too stiff compared to native myocardium	1: hiPSC‐CMs, non‐primary	1: 2D monolayer on PDMS stretchable chip, directionally aligned by stretch	1: 24 h for adhesion and 24 h for the stretch test	4
Renal epithelial cell volume regulation under hyperosmotic stress requires confluence and intact tight junctions; cells activate Na‐K‐2Cl cotransporter (NKCC) to restore volume after shrinkage, demonstrating a complex osmotic/mechanical response dependent on cell‐cell junctions [303].	Live‐cell volume measurements, pharmacological inhibition (NKCC), osmotic challenges, and immunofluorescence of tight junction proteins	Hyperosmotic stress	1: Controlled, physiologically relevant osmolarity shifts	0: cell line‐based renal epithelium	1: 2D confluent monolayers, epithelial tissue model	0: Short‐term (3 h)	2

CO‐CULTURE SCALE							
Summary and Key Finding	Evaluation Method	Stimulus	Mechanical Representativeness	Cell Source	Cellular Organization	Time Scale	MFS
Fibroblasts on stiff (50 kPa) elastic hydrogels mimicking fibrotic tissue show increased activation (spreading, type I collagen, cadherin‐11); conditioned media from macrophages did not alter this mechanical effect; direct co‐culture with M2 macrophages overrode hydrogel mechanics, activating fibroblasts even on soft viscoelastic hydrogels that normally suppress activation; IL6 signaling inhibition nullified this effect, restoring fibroblasts to a quiescent state [293].	Fibroblast morphology (spread area, circularity), immunostaining for type I collagen and cadherin‐11, hydrogel rheology, conditioned media vs. direct co‐culture, inhibitor studies (actin, myosin II, ROCK, TGFβ, IL6)	Substrate stiffness	2: Hydrogel stiffness and viscoelasticity tuned to match normal (1 kPa VE) vs. fibrotic (50 kPa elastic) lung mechanics	1: Human lung fibroblasts (hTERT T1015 cell line) and THP‐1 monocyte‐derived macrophages (polarized to M0, M1, M2)	1: 2D hydrogel; fibroblast monoculture, indirect (conditioned media), or direct macrophage co‐culture	1: The actual experimental co‐culture exposure on the hydrogels is 48 h	5
Endothelial cell TGF‐β1 expression under shear stress in a co‐culture model alters smooth muscle cell phenotype and MMP production (e.g., MMP‐2), linking endothelial signaling with vascular remodeling [304].	siRNA transfection to suppress TGF‐β1 in ECs; Western blotting for SMC contractile proteins (α‐SMA, calponin); RT‐PCR for MMP‐2 and MMP‐9 gene expression	Shear stress	1: Shear stress is in physiological relevant range	2: Primary human endothelial cells and vascular smooth muscle cells in co‐culture	2: 3D co‐culture	2: Up to 21 days, 72 h flow exposure for observing phenotypic and gene expression changes	7
Cyclic strain reduces neural stem cell (NSC) proliferation (quiescence induction) but does not alter lineage commitment; NSCs and young astrocytes align parallel to cyclic strain, unlike most mammalian cells (which align perpendicular); neurons lack mechanoresponsiveness, but astrocytes provide a mechanoprotective effect, shielding neurons from mechanical strain [305].	Live‐cell imaging, BrdU proliferation assay, immunocytochemistry (Sox2, GFAP, Tuj1, nestin, tubulin, actin, vimentin), confocal microscopy/3D reconstruction, cytoskeletal orientation analysis (image analysis), RhoA activation (LPA), neuron‐astrocyte co‐culture with variable ratios	Cyclic uniaxial strain	1: Mechanical strain mimics physiological pulsatile brain tissue deformations (∼up to 30% strain from vascular pulsation)	1: Primary rat neural stem cells (embryonic hippocampus), primary cortical neurons (E18–19 Wistar rats), postnatal astrocytes (P1–P3 Wistar rats)	1: 2D culture on PDMS elastomer chambers; NSCs in monoculture or coculture with neurons/astrocytes	1: Short‐term strain (24 h) for proliferation and cytoskeletal assays; longer‐term (5 days) during differentiation under cyclic stretch	4

3D CULTURES							
Summary and Key Finding	Evaluation Method	Stimulus	Mechanical Representativeness	Cell Source	Cellular Organization	Time Scale	MFS
Intestinal organoids spontaneously transition from spherical cysts to budded structures through a mechanical buckling instability; local differences in proliferation rate and apical contractility generate compressive stress that bends the epithelium inward, initiating budding; the work demonstrates that mechanical feedback between growth, curvature, and cytoskeletal tension can explain organoid shape transitions without requiring chemical patterning cues [306].	3D confocal live imaging, quantitative morphometric analysis, laser ablation (to estimate epithelial tension), pharmacological manipulation (actomyosin inhibitors, proliferation modulators), finite‐element and vertex‐based mechanical modeling	Scaffold stiffness	1: The model mimics epithelial mechanics of intestinal morphogenesis, but no active cues	2: Primary murine intestinal crypt stem cells (Lgr5^+^) from the mouse small intestine	2: 3D crypts or single ISCs are encapsulated in Matrigel or PEG hydrogels	1: Imaging 4 days post‐embedding	6
Photocrosslinking of GelMA/HAMA hydrogels on PTFE reduces surface friction (long‐lasting), enhances lubrication via water exudation, lowers shear strains, and promotes superior ECM (collagen II, aggrecan) accumulation under mechanical loading compared to glass‐crosslinked constructs [307].	Tribological testing (custom tribometer, friction coefficient µkin), AFM, DSC, digital image correlation (Exz strain), immunofluorescence (collagen II, collagen I, aggrecan), biochemical assays (DNA, GAG, wet weight, compressive modulus), live/dead viability staining, gene expression	Dynamic cyclic compression and biaxial mechanical loading	2: Stimulus mimics physiological cartilage mechanics; physiological joint‐like conditions (30% compressive strain, 1 Hz, 1 mm shear amplitude, 1 h/day)	2: Primary human articular chondrocytes from adult donors	2: 3D hydrogel culture	2: Preculture 14 days for PCM formation, followed by 14 days of dynamic biaxial loading	8
Intestinal organoids derived from mouse intestinal crypts display mechanochemical bistability during morphogenesis: feedback between lumen pressure (osmotic forces) and cortical actomyosin tension produces two stable morphological states (e.g., bulged vs budded). The timing of lumen volume changes controls transitions, showing history‐dependent mechanical regulation of morphogenesis; mechanosensation stabilizes these states [308].	Combination of 3D vertex modeling, live‐cell imaging (LifeAct‐GFP, Myh9‐GFP), laser ablation (lumen pressure release), pharmacological modulation (blebbistatin, PGE2), and morphometric analysis. Quantitative model–data comparison of shape, lumen volume, and myosin intensity ratios	Passive deformation	2: Organoids recapitulate physiological crypt–villus mechanics and cell fate patterning; lumen pressure measured via ablation provides direct biophysical validation, though simplified geometry compared to in vivo intestine	2: Crypts isolated from the small intestine of LifeAct‐GFP and Myh9‐GFP mice (primary murine intestinal epithelial cells)	2: 3D Matrigel organoids with fluid‐filled lumens mimicking intestinal architecture	1: Morphogenesis tracked over several days (up to 5 days post‐seeding), with live imaging intervals (20–30 min for dynamic transitions).	7
The study introduces a mechanically dynamic 3D intestinal organoid culture system by applying cyclic uniaxial stretch (8% strain, 0.2 Hz) to crypt‐derived organoids; stretching significantly increased organoid size, crypt number, and proliferation of Lgr5^+^ and Sox9^+^ stem cells, activating the Wnt/β‐Catenin pathway even in the absence of R‐spondin1; this demonstrates that mechanical stretch alone can sustain stemness and regeneration in intestinal organoids [309].	Immunofluorescence (Ki67, Sox9, Mmp7), immunohistochemistry, H&E staining, RNA‐Seq, qRT‐PCR, paraffin section histology, live imaging, GSEA, KEGG pathway analysis	Cyclic uniaxial stretch	2: Frequency and amplitude mimic intestinal peristaltic strain; strain applied using Flexcell Tissue Train system (optimized at 8% strain and 0.2 Hz); comparison of physiologically relevant stiffness using Matrigel and reproducible synthetic PEG as encapsulation	2: Primary mouse intestinal crypt stem cells (Lgr5^+^) isolated from C57BL/6J or Lgr5‐eGFP‐IRES‐CreERT2 transgenic mice	2: 3D Matrigel–collagen (50/50 v/v) organoids in Flexcell system undergoing cyclic uniaxial stretch	1: 3 days incubation, followed by stretching for 3 days, and harvested on day 7	7

ORGANOTYPIC TISSUE MODELS							
Summary and Key Finding	Evaluation Method	Stimulus	Mechanical Representativeness	Cell Source	Cellular Organization	Time Scale	MFS
3D intestinal organoids maintain lumen symmetry and correct apico‐basal polarity through the interplay of apical actomyosin tension and basal ECM anchoring; when actomyosin contractility or matrix stiffness is disrupted, the lumen collapses or becomes irregular, and cell polarity markers become mislocalized; this demonstrates a mechanochemical feedback that stabilizes epithelial organization during early morphogenesis [159].	Immunostaining (E‐cadherin, ZO‐1, F‐actin, Lysozyme, Lgr5), confocal microscopy and live imaging, laser ablation for apical tension, and pharmacological inhibition (blebbistatin, cytochalasin D)	Hydrostatic pressure	1: Hydrostatic lumen pressure and apical junctional tension regulate epithelial lumen morphology in a simplified epithelial cyst model	0:MDCK‐II canine kidney epithelial cell line (WT, CLDN‐KO and ZO1/ZO2‐KO)	2: 3D Matrigel organoids showing lumen formation and polarity establishment	1: 4‐5 days until lumen formation and establishment of epithelial polarity	
Biomimetic culture of precision‐cut human ventricular myocardial slices (from explanted failing hearts) in custom biomimetic cultivation chambers (BMCCs) that provide physiological preload, compliance, continuous electrical excitation and medium agitation preserves structure and function long‐term: slices reach a stable contractile state that can be monitored up to 4 months (≈2 000 000 beats); the system enables repeated functional testing (force, refractory period, action potential) and transcriptomic / histological analyses and is suitable for chronic drug‐safety testing [310].	Continuous contractility monitoring via magnetic spring‐wire sensor in BMCCs; isometric organ‐bath twitch‐force measurements; intracellular membrane‐potential recordings (sharp electrodes); stimulation/control electronics recording; histology / immunohistochemistry (H&E, α‐actinin, connexin‐43, N‐cadherin, vimentin, SMA); confocal imaging and morphometry (sarcomere, T‐tubule metrics); RNA‐Seq (polyA, STAR/HTSeq/DESeq2 pipeline) at multiple culture timepoints; pharmacological perturbations (isoprenaline, pentamidine, dofetilide); refractory‐period assays	Cyclic contraction under defined load	2: Combined preload (set to 1 mN), continuous rocking motion and continuous electrical pacing (at 0.2 Hz); pacing frequency is purposely set low and is therefore non‐physiological in rate, but the type of cue (tension + contractile cycling + electrical pacing) is physiologically relevant cardiac‐mechanical loading	2: Human adult left‐ventricular myocardium obtained from explanted failing hearts (transplant specimens; patients gave informed consent); up to 30 slices prepared per specimen; 18 patients used in experiments	2: Precision‐cut 300‐µm myocardial tissue slices (≈5 × 5 mm^2^ muscle area) glued to plastic triangles and mounted in BMCCs providing an elastic mount + preload, continuously paced and agitated in a standard CO_2_ incubator	2: Functional monitoring up to 4 months; structural, transcriptomic, and histological analyses commonly reported up to ~35 days	8
Application of biomimetic electromechanical stimulation (physiological preload and electrical pacing) preserves the structure, function, and transcriptional profile of adult myocardial slices from rat, rabbit, and human hearts; a preload equivalent to sarcomere length (SL = 2.2 µm) optimally maintains contractility, Ca^2^^+^ handling, t‐tubule structure, and gene expression at 24 h (rat, human HF slices); rabbit slices remained functional for 5 days under the same conditions; unloaded slices rapidly dedifferentiate; electromechanical stimulation prevents fibroblast overgrowth and maintains energy balance and adult cardiac phenotype [311].	Functional assays: contractility measurements (force transducer), Ca^2^^+^ imaging with Fluo‐4 and optical mapping, multielectrode array conduction measurements, β‐adrenergic response assays, and arrhythmia induction. Structural assays: immunohistochemistry/confocal microscopy for sarcomeric and t‐tubule markers (Cav3, α‐actinin, TOM20, connexin 43, vimentin). Ultrastructure by TEM. Molecular assays: RNA‐Seq (gene set enrichment), ATP and metabolite quantification (HPLC), and CellTiter viability assay	Uniaxial stretch and cyclic deformation	2: The type of loading (preload + paced contraction) is physiologically relevant; the rates used are sub‐physiological for the species (intentionally lowered to support viability)	2: Adult myocardial tissue: rat ventricles (healthy), rabbit ventricles (healthy), and human left‐ventricular myocardium from explanted failing hearts (heart‐transplant donors)	2: 3D myocardial tissue slices (≈300 µm thick) with preserved multicellularity and extracellular matrix, cultured in custom‐made perfused chambers providing preload and field stimulation. Unloaded control slices were cultured on a liquid–air interface (Transwell inserts)	1: 24 h (rat and human HF slices) and 5 days (rabbit slices) under continuous electromechanical stimulation	7

### 7.3 Limitations of Current In Vitro Studies and the MFS

In vitro assays offer precise control over variables, but they inherently simplify the native environment. Real tissues involve *multiple cell types* (fibroblasts, macrophages, etc.) interacting over time and overwriting mechanical responses [293, 294]. Processes such as mechanoadaptation, recursive feedback signals, and long‐term tissue remodeling occur over extended periods that are difficult to reproduce in standard in vitro systems. This significantly limits the predictive value of many assays for in vivo results [294]. In addition, experimental conditions vary considerably between studies, e.g., with regard to cell source, matrix composition, stiffness, loading regimes, and culture duration. This often leads to limited reproducibility and direct comparability. If mechanical stimulation is not adequately adapted to physiological conditions, experimentally induced responses may be artifactual or biologically misleading. Importantly, mechanobiological responses are often nonlinear and threshold‐dependent rather than continuously dose‐dependent, as cells can buffer weak mechanical inputs until critical activation thresholds are exceeded. This leads to disproportionate downstream effects on signaling pathways, ion flux, cytoskeletal organization, or gene regulation. Consequently, seemingly similar experimental systems can produce substantially different biological outcomes depending on the precise mechanical context and loading history.

**FIGURE 6:**
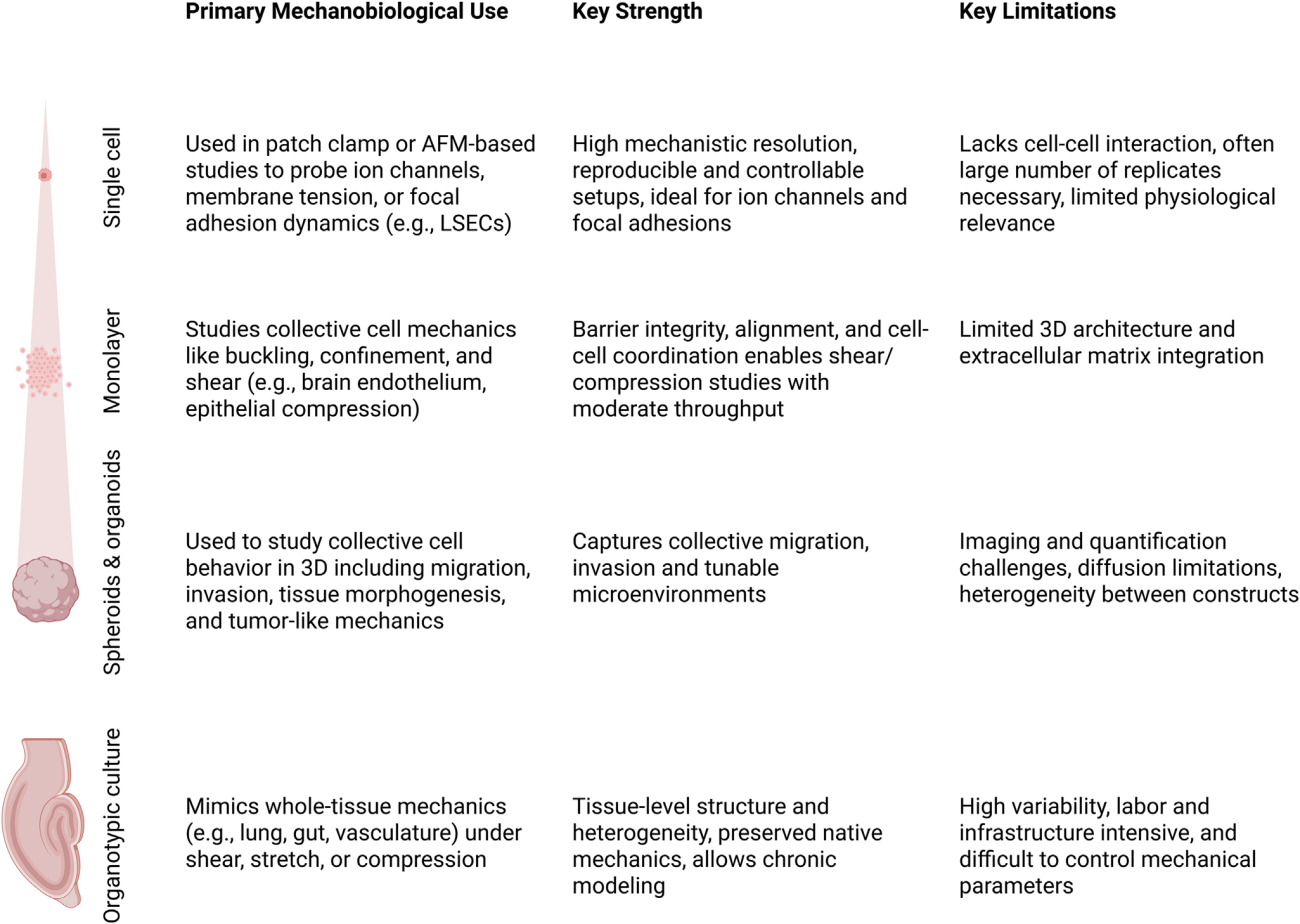
Comparative overview of in vitro mechanobiological model systems across different complexity levels with their strengths and limitations.

Simplified systems such as conventional 2D cultures and single‐cell assays further lack important physiological features, including multicellular organization, 3D matrix architecture, blood flow, innervation, hormonal signaling, and immune interactions. Single‐cell measurements are also highly sensitive to factors such as cell morphology, proliferation state, and attachment conditions, often requiring a large number of replicates to ensure robust, reliable results. Although advanced systems such as organoids, co‐cultures, organ‐on‐chip platforms, and ex vivo tissue models improve physiological relevance, they still face limitations such as donor variability, lack of standardization, imaging difficulties, and limited long‐term stability. Figure 6 summarizes the major experimental model systems discussed in this review with regard to their biological and structural complexity, strengths, weaknesses, and characteristic mechanical influences.

The proposed MFS has some important limitations. First, it has not yet been calibrated against historical in vivo or clinical outcomes and should therefore not be interpreted as a predictive translational metric, but rather as a structured framework for the comparative description of mechanobiological fidelity across experimental systems. Second, the equal weighting of the four MFS dimensions was intentionally selected as a transparent starting point, although the relative importance of each dimension is likely context‐dependent and not compensatory. For example, physiological loading in vascular systems may be of central importance, while multicellular organization or long‐term adaptation may be more critical in developmental or fibrotic models. Accordingly, a higher MFS should be interpreted as an indicator of greater contextual fidelity rather than a guarantee of translational success. The MFS is particularly useful for evaluating advanced mechanobiological systems designed to mimic physiological tissue environments, including 3D cultures, organoids, co‐cultures, and organ‐on‐chip platforms. However, its relevance may be limited in highly reductionist systems primarily aimed at mechanistic isolation, such as single‐cell assays or exploratory 2D screening studies. Future studies should therefore validate the MFS against defined translational benchmarks and in vivo reference datasets.

While no clear trend can be identified across the average lowest sub‐score of each scale, a recurring limitation of many analyzed studies is the tendency to draw conclusions about in vivo relevance without adequately demonstrating translational relevance, be it through mechanistic reasoning, a suitable cellular context, or comparative validation. Several studies, including those using immortalized cell lines under artificial conditions, extrapolate results to in vivo scenarios without demonstrating that the observed responses also apply outside the simplified in vitro setup. Frequently, important physiological parameters such as cell confluence, tissue‐specific stiffness zones, or native‐like flow and pressure environments are either ignored or inadequately replicated, yet the results are presented in the discussion as directly predictive. Even more concerning is the lack of distinction between mechanistic and functional translational insights, where a molecular response is often implicitly assumed to reflect tissue‐level behavior without validation. This oversimplification not only undermines the credibility of the conclusions but also blurs the line between useful in vitro reductionism and unjustified overinterpretation. As our comparative table shows, the studies that clearly define their biophysical cues, use primary cells, or validate findings under disease‐relevant or in vivo‐mimicking conditions stand out in their clarity and translational value, underscoring the need for more disciplined, transparent modeling approaches in mechanobiology.

Larger experimental scales tend to show higher average MFS values. However, experiments with well‐defined, physiologically relevant, and context‐dependent forces can still reproduce in vivo responses, particularly in mechanically active tissues. Advanced soft tissue models offer predictive power when mechanical inputs and cellular contexts are appropriately chosen. Numerous studies consistently demonstrate that cells thrive only in stiffness ranges that match their native tissue: fibroblasts adapt within physiological soft tissue ranges [293], and chondrocytes and muscle cells, respectively, spread and differentiate only on sufficiently stiff matrices [312]. This principle of stiffness adaptation has been confirmed in vivo, where deviations due to fibrosis, scarring, or injury lead to maladaptive cellular responses.

## 8 Discussion

Mechanobiology provides crucial insights into how cells perceive and respond to physical cues. This contributes to the development of regenerative strategies and the design of responsive biomaterials. Advances in in vitro systems have enabled increasingly precise control over cell fate, alignment, and collective behavior. However, challenges remain in replicating the full physiological and pathological context in which these mechanical signals are interpreted. This comparative analysis highlights several trends worthy of attention, which are discussed in the following sections.

### 8.1 Mechanobiological Response is Time‐Dependent

Many important physiological processes occur over extended periods or in complex mechanical environments that in vitro systems only partially replicate. Mechanoadaptation (i.e., the gradual adjustment of cellular structure, function, or signaling in response to sustained or repeated mechanical cues) and recursive signaling loops (i.e., the bidirectional interaction of materials and biological environments) often require chronic or cyclic stimulation over extended periods of time, which exceeds the duration of most standard assays [15, 311]. Immunomodulation, fibrotic remodeling, and tissue degeneration are also characterized by temporally layered and multicellular interactions that simple models struggle to simulate. Mechanobiology is not static—cellular responses vary depending on niche, timescale, and mechanical history—making long‐term or in vivo validation and the careful interpretation of how short‐term responses relate to long‐term outcomes essential for meaningful interpretation.

Most studies still treat the mechanical microenvironment as if a single scalar stiffness parameter suffices, when in reality biological matrices are profoundly viscoelastic [76, 77, 78]. They dissipate energy at rates comparable to the timescales of cellular signaling pathways. A gel with identical instantaneous stiffness can yield completely different cell fate outcomes depending solely on stress‐relaxation kinetics. Most mechanobiology readouts still mention simple numbers  as if these were independent of frequency, history, or strain amplitude. This discrepancy between *rheology* and *biology* is one of the most underestimated causes of poor reproducibility.

Furthermore, mechanical conditioning is not time‐neutral—cells possess a mechanical memory. A two‐hour and a seven‐day assay differ not only in duration but also examine different dynamic states of the cell's mechano‐epigenetic landscape. To distinguish “cause” from “pretreatment,” the load history must be explicitly considered, not just the load intensity.

In addition, bulk tissue mechanics is not only solid‐like. Interstitial fluid pressure, convective flow, poroelastic coupling, and local osmotic shifts modulate adhesion forces and molecular crowding on the same timescales as mechanotransduction. Cell contraction, for instance, deforms not only polymer chains but also expels water, concentrates soluble factors, and alters gradients. However, many platform materials (e.g., non‐porous PEG) cannot reproduce solvent migration or permeability changes that occur in native ECM under load. Neglecting this fluid‐solid coupling erases an entire class of time‐dependent feedbacks that shape mechanosensitive signaling in vivo.

### 8.2 Complexity Doesn't Always Equal Predictivity

Complexity alone is not a reliable indicator of physiological accuracy. Liver organoids cultured in stiff, cross‐linked hydrogels do exhibit strong expansion via YAP activation—however, this stiffness more closely mimics fibrotic or neoplastic liver tissue than the compliant microenvironment of development [313, 314]. This example illustrates a broader concern: without the appropriate context to in vivo conditions and fundamental understanding of the origin of biomechanical parameters and corresponding cellular responses, even structurally advanced systems may fail to replicate the physiological state of interest. Likewise, cellular responses that are commonly interpreted as positive indicators of compatibility may be context‐dependent: proliferation is often taken as evidence of a supportive environment, whereas increased proliferation together with migration may also indicate that cells are attempting to remodel, escape, or adapt to a mechanically unsuitable microenvironment. Conversely, relatively simple 2D or single‐cell models can provide highly predictive results if isolated biophysical cues closely mimic in vivo conditions [33, 301, 302].

### 8.3 Cell‐Cell Interactions Play a Crucial Role in Physiological Relevance

One of the major limitations of in vitro models is the inadequate or overly simplistic representation of cell‐cell communication. Many widely used systems, such as single‐cell assays or non‐confluent planar monolayers, neglect the mechanical and biochemical interplay between neighboring cells that are crucial for tissue‐level coordination. Mechanosensitive signaling pathways—including the YAP/TAZ and RhoA pathways—are modulated not only by substrate stiffness but also by interaction mechanisms, cell junction tension, and the collective integrity of the cytoskeleton [157, 158, 173, 183, 258].

This deficiency proves particularly problematic in systems where mechanical decisions are made cooperatively, such as cell/tissue development, immune responses, or fibrotic remodeling. Experiments that isolate cells from their neighbors may fail to predict emergent properties or collective behaviors in vivo. Including appropriate multicellular organization is often more impactful for translational relevance than simply increasing structural or dimensional complexity.

Embedding cells in a scaffold or matrix typically creates a mechanically more active environment with integrin anchorage points and ample space for cell spreading, leading to the activation of YAP/TAZ. In contrast, encapsulation keeps cells in a mechanically passive environment, often inhibiting YAP/TAZ unless the material properties are altered, and preventing cell‐cell interactions. The choice between these approaches depends on the application: if robust cell mechanotransduction and maturation are desired (e.g., in bone tissue engineering), scaffold embedding may be advantageous; if cell quiescence or immunoisolation is required (e.g., for cell therapies), encapsulation may be beneficial [183, 259]. Understanding how each method affects mechanosensitive pathways allows bioengineers to design the cell delivery format that best meets their mechanobiological objectives.

### 8.4 Choosing the Right Cell Source for Translational Fidelity

There is an important distinction between cell lines, stem cell‐derived organoids, and tissue‐derived organoids. Immortalized cell lines often display hyper‐plastic responses and resemble a fetal‐like mechanosensitive state, limiting their predictive value for adult pathophysiology. Stem‐cell‐derived organoids improve upon this by recapitulating developmental programs, but they too tend to reflect immature tissue mechanics. In contrast, tissue‐derived organoids preserve niche‐specific architecture and mature mechanosensory signatures, making them uniquely powerful for studying adult disease contexts such as fibrosis or cancer. Incorporating these systems into translational models therefore enhances both the mechanocompatibility and the physiological fidelity of in vitro research [306, 308, 315].

### 8.5 Cellular Response Analyses can Extend From the Molecular Level to the Tissue Level

Mechanotransduction is an inherently hierarchical and multi‐scale process, ranging from molecular force measurement to tissue remodeling and physiological adaptation. The aim of this review article was to apply this hierarchical and effect‐driven framework not only to biomaterial design and cellular model systems themselves, but also conceptually to the experimental methods used to assess cellular responses. Although the review is not strictly structured according to this hierarchy, many analytical techniques can nevertheless be categorized along a scale‐driven continuum that reflects the different biological levels at which mechanical information is generated, transmitted, and interpreted.

At the nanoscale, methods such as FRET‐based tension sensors, super‐resolution microscopy/iPALM, and TIRF enable the selective resolution of the immediate cell–material interface and provide insights into force transmission in the piconewton range, the organization of focal adhesions, and the spatial stratification of integrins, adaptor proteins, and actin within adhesion complexes [261, 266]. At the cellular and mesoscale levels, confocal and live‐cell imaging approaches capture emergent cytoskeletal and junctional remodeling, mechanosensitive signaling events such as the nuclear translocation of YAP/TAZ, and dynamic adaptations to mechanical stimulation, including strain‐rate‐dependent reorientation of stress fibers and MAPK activation under cyclic loading. In contrast, ultrastructural and tissue‐specific methods such as TEM, SEM, histology and tissue imaging primarily contextualize these responses in the context of broader architectural and remodeling processes, including matrix organization, fibrosis and implant integration, but mainly serve to analyze fixed endpoints rather than measure dynamic signals [269].

Consequently, the methodological landscape itself reflects the hierarchical propagation of mechanical information across different biological scales and underscores that mechanotransduction cannot be adequately interpreted at a single spatial or functional level. At the same time, this perspective highlights important translational limitations of current mechanobiological models, including the discrepancy between often static in vitro environments and the dynamic loading conditions in vivo, as well as the trade‐off between spatial and temporal resolution and live‐cell compatibility in different methodological platforms.

### 8.6 Pathological Models Offer Functional Validation

Validation under disease‐relevant conditions is another critical step toward translation and often not considered. Mechanosensitive responses observed under baseline or non‐diseased experimental conditions may not behave identically in pathological contexts. Therefore, models that recapitulate disease‐specific mechanical environments—such as fibrotic matrices, tumor‐like stiffness, or chronically inflamed conditions—are essential for confirming mechanistic relevance if the in vivo response is well studied and comparable. These pathophysiological models not only serve as functional tests of mechanobiological hypotheses but often reveal novel aspects of signaling that are masked in homeostatic conditions.

### 8.7 Fibroblasts Play an Important Role in Mechanobiological Studies

Among the cell types studied, fibroblasts stand out for their robust mechanoadaptive behavior. Unlike many cells that fail to thrive on very soft substrates, fibroblasts can dynamically tune their internal stiffness to match that of their environments [297]. This adaptability highlights their central role in wound healing and fibrosis, where they sense changes in matrix rigidity and respond with cytoskeletal reinforcement, stress fiber formation, and altered gene expression. Compared to neurons or chondrocytes, which exhibit narrow stiffness optima and often lose function outside of native‐like conditions, fibroblasts demonstrate remarkable plasticity. In addition, fibroblasts are comparatively easy to maintain and expand in vitro, tolerate a broad range of culture conditions, and exhibit reproducible responses to mechanical perturbation, making them particularly attractive as experimental mechanobiology models. This makes them both valuable as a model system to study mechanotransduction and clinically important as drivers of fibrosis and pathological stiffening. Interestingly, macrophages override fibroblast mechanosensing, highlighting the importance of multicellular cultures [293].

### 8.8 Mechanocompatibility and Scaling Defines Physiological Relevance

Besides biological aspects, material properties play a crucial role in shaping cell behavior. Modern biomaterials can replicate viscoelasticity, anisotropy, and dynamic cues, but the complex, actively remodeling mechanics of native tissues on a multiscale level are rarely matched exactly. These mismatches—especially in magnitude and spatio‐temporal organization—affect force transmission and cell reorganization. These differences affect how forces are transmitted and how cells reorganize in response to them. Furthermore, material modification strategies—such as altering osmolarity or crosslinking density—can unintentionally disturb mechanosensitive responses by changing membrane tension, ion homeostasis, or cytoskeletal dynamics [292].

Mechanocompatibility in experimental setups should be considered a context‐dependent attribute similar to in vitro compatibility—as introduced with the MFS. It is not defined solely by stiffness, but by how well the material integrates with cell‐cell junctions, ECM interfaces, and the broader mechanical architecture of the tissue. The same material may support homeostasis in one system but induce pathological activation in another, depending on its ability to sustain appropriate connectivity and signaling fidelity. One example of misinterpreted mechanical parameter identification in biomaterials as well as tissue is the conflation of bulk mechanical properties—such as the overall stiffness of a hydrogel—with micromechanical properties like the local stiffness of individual polymer strands. This distinction is crucial because single cells and cell aggregates often respond to micro‐level mechanical cues that cannot be accurately captured by bulk measurements of the material alone. At the same time, bulk mechanical properties may still dominate tissue‐ and organ‐scale behavior. Failing to distinguish between these scales can therefore lead to misleading conclusions regarding cellular mechanosensitivity.

### 8.9 Mechanomodulation Enables Staged Engineering of Cell States

The ability to selectively control differentiation or migration is a major practical benefit: if cells are first driven into the desired lineage and only then exposed to a tuned, more physiological'stiffness', cultures can expand faster initially and subsequently mature under conditions that better recapitulate native biophysical constraints for in vivo‐like organization [316, 317]. This stepwise approach effectively decouples “growth‐optimal” from “fidelity‐optimal” mechanics—a concept rarely addressed explicitly in mechanobiology but widely used in bioprocess engineering. Early‐phase softening or stiffening can act as a lever to accelerate expansion or promote early lineage priming, while later mechanical adjustment restores physiological ranges to re‐establish proper ion channel gating, cytoskeletal homeostasis, and metabolic setpoints. Importantly, this does not require structural complexity: even simple 2D systems can be engineered to switch between growth‐optimized and fidelity‐optimized stiffness states via modulatable matrices, degradable crosslinks, or osmolarity shifts. Such staged mechanomodulation therefore represents a design principle [317, 318].

### 8.10 Translational Relevance Depends on Intended Design

Emerging platforms—such as perfusable organoids, immunocompetent co‐cultures, and dynamic in vivo models like the chorioallantoic membrane [2] ‐ offer new opportunities to recapitulate physiological mechanics and multicellular interactions. However, one principle remains consistent across platforms: translational success depends not just on complexity, but on how completely the model reproduces the mechanical, biological, and temporal architecture of the tissue being studied.

The cytoskeleton and adhesion complexes act as universal mechanosensors linking extracellular stiffness to cellular behavior. In fibroblasts, increased stiffness promotes the formation of actin stress fibers and integrin‐mediated signaling, while in neurons, substrate stiffness modulates the dynamics of focal adhesions, the activity of Ca^2^^+^ channels, and downstream signaling pathways such as EGFR/PI3K/AKT [295, 297]. These mechanotransductive cascades, in turn, determine functional outcomes such as cell survival, excitability, and regenerative potential. Despite differing optimal ranges among cell types, the general trend is clear: cells are tuned to thrive within the stiffness range of their native tissue, and deviations from this range lead to maladaptive phenotypes.

From a translational perspective, these findings lead to a key insight: the mechanical microenvironment is not a passive scaffold but an instructive cue that dictates cell fate, function, and therapeutic response. Healthy tissues are soft and compliant, while injury, fibrosis, and scarring typically increase stiffness, leading to pathological adaptations. For neurons, this may mean loss of viability or dysregulated excitability; for fibroblasts, excessive contractility and fibrosis; and for cartilage, accelerated degeneration under high friction. Consequently, biomaterial design and drug testing must carefully replicate the physiological mechanics of target tissues. Without stiffness‐matched environments, in vitro studies risk misrepresenting in vivo outcomes, highlighting why mechanobiology is central to tissue engineering, regenerative medicine, and translational neuroscience.

## 9 Conclusion

Translational prediction in mechanobiology depends on mechanocompatibility rather than complexity: cellular models must capture physiological inputs, context, and cell source. In this respect, tissue‐derived organoids are uniquely valuable, as they preserve the mechanosensitivity of mature tissues, while stem‐cell‐derived organoids provide insights into developmental programs and immortalized cell lines often reflect fetal‐like states. In many studies, limitations and correction factors are often ignored. Because mechanobiological responses are time‐dependent and shaped by mechanical history, matrix viscoelasticity, fluid‐solid coupling, and bidirectional cell‐material interactions, short‐term readouts must be interpreted in relation to long‐term adaptation and in vivo outcomes. Moreover, cellular responses such as proliferation, migration, differentiation, or YAP activation are not inherently positive or predictive, but must be evaluated against the intended physiological or pathological context, including multicellular organization, disease‐specific mechanics, and the relevant tissue scale. In many studies, limitations and correction factors are often ignored. Combining these fundamental limitations with contextual mechanocompatibility will be key to building next‐generation models that not only explain cellular mechanosensing but also reliably predict in vivo outcomes.

## Author Contributions

J.F., M.P., and M.P.K. contributed to conceptualization, investigation, methodology, validation, visualization, data curation, manuscript drafting, and manuscript review and editing. N.H. contributed to conceptualization, investigation, methodology, and manuscript review and editing. T.R. contributed to conceptualization, methodology, manuscript drafting, and manuscript review and editing. K.L., R.K., and M.T. contributed to investigation, methodology, validation, manuscript drafting, and manuscript review and editing. G.S. contributed to manuscript review and editing, and supervision. G.A.H. contributed to manuscript review and editing. J.F. was additionally responsible for formal analysis, project administration, funding acquisition, and correspondence. M.P., M.P.K., G.S., and J.F. provided supervision. All authors reviewed and approved the final version of the manuscript.

## Conflicts of Interest

The authors declare no conflicts of interest.

## Data Availability

Data sharing not applicable to this article as no datasets were generated or analysed during the current study.
